# Pain Neuroscience Education and Resistance Training in Women With Fibromyalgia: A Randomized Control Pilot Study

**DOI:** 10.1155/prm/7550108

**Published:** 2025-07-10

**Authors:** Álvaro-José Rodríguez-Domínguez, Manuel Rebollo-Salas, Raquel Chillón-Martínez, Melania Cardellat-González, Laura Blanco-Heras, José-Jesús Jiménez-Rejano

**Affiliations:** ^1^Department of Health and Sports, Pablo de Olavide University, Seville, Spain; ^2^Department of Physiotherapy, University of Seville, Seville, Spain

**Keywords:** exercise therapy, fibromyalgia, pain neuroscience education, physical therapy modalities

## Abstract

**Objective:** The objective was to compare the effectiveness of a combined pain neuroscience education and resistance training program (PNE + RT) with that of a combined aerobic and flexibility exercise program (AE + FE).

**Design:** A randomized pilot study was conducted in women with fibromyalgia.

**Methods:** Thirty-one women with fibromyalgia were randomized into the experimental group (PNE + RT, *n* = 15) and the usual care group (AE + FE, *n* = 16). Both groups carried out the intervention 3 days a week for 12 weeks. Primary outcomes were pain intensity, disability, and symptoms related to central sensitization (CS). Among them, pain intensity was considered the main primary endpoint for statistical analysis and interpretation. Secondary outcomes were pressure pain threshold (PPT), maximum handgrip strength (MHS), and stiffness.

**Results:** Statistically significant between-group differences were found in favor of PNE + RT group for short-term pain intensity (*p* < 0.05) and PPT trapezius (*p* < 0.05). PNE + RT also showed statistically significant within-group improvements in pain intensity (*p* < 0.01), CS-related symptoms (*p* < 0.01), PPT quadriceps (*p* < 0.01), and MHS of the left hand (*p* < 0.01). Disability improved significantly in both groups (*p* < 0.01). There were no significant changes in stiffness.

**Conclusion:** The PNE + RT program is more effective than the AE + FE program in improving pain intensity in the short term and PPT in the trapezius muscle in the long term. PNE + RT is also effective in improving disability, pain intensity, CS-related symptoms (short and long term), and left MHS and PPT in the quadriceps muscle (long term), although it is not more effective than AE + FE. The AE + FE program is only effective in improving disability. These findings are preliminary, and larger studies are needed to confirm the results.

**Trial Registration:** ClinicalTrials.gov identifier: NCT04855851

## 1. Introduction

Fibromyalgia (FM) is a chronic syndrome that causes widespread musculoskeletal pain, fatigue, sleep disorders, and disability [1]. In addition to generalized pain, its most characteristic symptom, FM is also associated with psychiatric and cognitive disorders and hypersensitivity to stimuli not related to the somatosensory system, such as photophobia and phonophobia [1, 2]. Its prevalence is estimated and is between 2% and 4% of the world's population, and the female population is the most affected [3, 4]. Although the etiopathological mechanisms are not exactly known, they seem to be related to central sensitization (CS) [1].

CS is defined as “amplification of neural signaling within the central nervous system resulting in hypersensitivity to pain” [5]. The first time FM was related to SC was in 1994 [6]. The first studies that examined the pathophysiology of this phenomenon in humans were carried out precisely in patients with FM, as this was one of the first conditions in which predominantly central factors were identified [7].

The latest recommendations from European League Against Rheumatism (EULAR) for FM treatment show that the only intervention with a “strong” recommendation is in favor of physical exercise [8], although aspects related to dose and exercise modality are unknown. To date, the modality with the greatest support in the treatment of FM is aerobic exercise (AE) [9, 10], so the most commonly used consists of AE programs in combination with flexibility exercises (FEs) [11]. However, there is a growing body of evidence demonstrating the benefits of resistance training (RT) [12, 13], showing effects comparable to or superior to other exercise modalities in improving pain intensity, tender points, and functionality [14].

However, advances in neuroscience have led to the development of new therapeutic strategies aimed at reconceptualizing pain through a treatment method called pain neuroscience education (PNE). Several systematic reviews with meta-analysis demonstrate that this intervention can improve pain intensity in people with FM [15, 16], but in combination with a multimodal treatment that includes exercise therapy, it could be effective in improving pain, functionality, anxiety, and depression [15]. We have not found any study that has analyzed the effects of RT combined with PNE in patients with FM. Our hypothesis is that this combination will be more effective than the usual standard, which usually consists of AE and FE, with medication according to symptoms. Therefore, the objective of this study was to evaluate the effects of PNE in combination with RT (PNE + RT) compared to the AE combined with FE (AE + FE) and to continue with the usual medication. Hence, this study has an exploratory character, as it compares two different intervention protocols.

## 2. Methods

### 2.1. Study Design

This pilot study was designed as a prospective, single center, randomized controlled trial with a blinded evaluator. The study protocol was approved by the Clinical Research Ethics Committee of the University Hospital “Nuestra Señora de Valme” (Approval Number: C.I.: 0602-N-21). All procedures were carried out according to the 1964 Declaration of Helsinki and registered in the Clinicaltrials.gov database.

### 2.2. Participants

The patients were enrolled according to the following inclusion criteria: (1) women with a diagnosis of FM made by a rheumatologist according to the criteria of the American College of Rheumatology (ACR1990/2010/2016) [17–19], (2) 20–65 years of age, (3) duration of symptoms greater than one year, and (4) sufficient cognitive level to understand the procedure and indications received.

The following were exclusion criteria: (1) any health condition for which physical exercise was contraindicated, (2) the presence of any other disease that may explain the symptoms (as established by the ACR diagnostic criteria), (3) being under medical treatment not related to pain, (4) being under physiotherapy treatment related to pain, and (5) pregnant or postpartum women.

### 2.3. Procedures

Subjects were randomly assigned (José-Jesús Jiménez-Rejano) to one of the two intervention groups by simple randomization using Random Allocation Software. Measurements were performed by an external evaluator (blinded). The evaluator (Álvaro-José Rodríguez-Domínguez) did not have access to the group assignment. Furthermore, the assessments (Melania Cardellat-González and Laura Blanco-Heras) were conducted in separate sessions from the interventions, which ensured effective blinding. Internal checks were also conducted to verify that blinding was maintained throughout the study. All participants were informed (Manuel Rebollo-Salas), verbally and in writing, about the objectives and procedures of the study, and they signed an informed consent form before being included in the study.

Participants completed surveys for demographic and clinical data, which allows us to know if the groups were homogeneous at the beginning of the study (Manuel Rebollo-Salas). The primary outcomes were assessed using online questionnaires. For secondary outcomes, subjects were summoned to a medical center. These data were analyzed by another evaluator (Raquel Chillón-Martínez), who did not know the treatment to which each patient would be assigned. It was not possible to blind the therapists and patients to the intervention, as this is not possible in active treatments.

### 2.4. Measurements

#### 2.4.1. Main Outcomes

The main objective of this study was to evaluate the effectiveness of a PNE program in combination with RT on pain intensity, disability, and symptoms related to CS. These variables were assessed before the intervention (T0), at the end of the intervention (T1), 1 month later (T2), 3 months later (T3), and 6 months later (T4). The measurement instruments were as follows.

• Visual analog scale (VAS): This tool consists of a numerical scale from 0 (no pain) to 100 (more severe pain) that allows quantitative measurement of the intensity of pain experienced by the patient. This scale is considered one of the most sensitive tests to evaluate pain intensity, since it shows high inter-observer reliability [20].
• Fibromyalgia impact questionnaire-Spanish version (FIQ-S): This questionnaire of 10 items measures the self-reported quality of life of patients with FM. Its score ranges from 0 to 100, and higher scores indicate a lower quality of life and a greater disability. The Spanish version has been validated, showing to be reliable and sensitive to changes in health status and physical function in patients with FM [21].
• Central sensitization inventory (CSI): It is a tool for identifying patients with CS symptoms. Its score ranges from 0 to 100, and a score equal to or greater than 40/100 has been proposed as the cutoff point for the detection of symptoms of CS in chronic pain populations and has been shown to generate reliable and valid data to quantify the severity of these symptoms [22, 23].

#### 2.4.2. Secondary Outcomes

Secondarily, we evaluated the effects of this intervention on long-term sustainable changes in maximum handgrip strength (MHS), pressure pain threshold (PPT), and stiffness. For this purpose, the variables were analyzed before the intervention (T0) and 6 months later (T4). The following instruments were used.

• Algometer: This device measures the pressure applied to the skin in kg/cm^2^. An electronic pressure algometer (Wagner FPX™) was used, which has been shown to be highly reliable and interchangeable with the VAS paper version [24]. For the assessment, the participants were seated in a comfortable position while constant pressure was applied vertically to the points selected for the evaluation. Subjects were instructed to indicate when pressure became painful. The PPTs were recorded at two bilateral points. To avoid the risk of temporal summation, each site was assessed only once. The same order was used for all participants, starting with points on the right side in the following order: upper trapezius (in the middle third, over the suprascapular fossa) and quadriceps (in the distal third, near the knee); see Appendix 1 in the Supporting Information (SI).
• Dynamometer: MHS was recorded bilaterally with the hydraulic hand dynamometer (Jamar). This device has been validated and shows good reliability [25, 26]. The mean force was recorded over a set period of time (10 s) and the best performance of two tests (with 1 minute between each test) was used (Appendix 2 in SI).
• Myotonometer: Stiffness was assessed using Myoton Pro. This tool can measure muscle tone and stiffness with an intra-rater reliability with intraclass correlation coefficient of 0.87–0.98 and an inter-rater reliability of 0.76–0.86 [27]. The evaluation was performed in the same posture and location as the PPT (upper trapezius and quadriceps) (Appendix 3 in SI). The device measures mechanical oscillations using a short-duration mechanical impulse (15 ms) and a constant mechanical force (up to 0.6 N), obtaining the variable stiffness (N/m). Three measurements were performed with 10 min of rest between tests, and the average of all tests was used for statistical purposes [28].

### 2.5. Interventions

The participants were randomized into two groups, the experimental group (PNE + RT) and the usual care group (AE + FE). Both groups performed the exercise program at home three times a week for 12 weeks. A follow-up visit was made every 15 days. Both groups began with 5 min of warm-up, followed by 40–50 min of exercise program. To monitor treatment and ensure adherence, follow-up was conducted by telephone. An evaluator made a phone call twice a week to answer questions and ensure that treatment was followed.

#### 2.5.1. Experimental Group (PNE + RT)

The PNE sessions were structured following the “Explain Pain” manual, written by Moseley and Butler [29]. Each PNE session lasted approximately 60 min and was performed in person every 15 days (total of six sessions). The PNE sessions were conducted by a physiotherapist with training and experience in educational interventions for patients with chronic pain. After each session, support material related to the topics addressed was provided as home treatment. The PNE program addressed the following points: (1) conceptualization of the disease and importance of physical exercise, (2) relationship between injury and pain, (3) basic physiology of pain, (4) concept of CS, (5) influence of psychosocial factors, (6) neuroplasticity, and (7) concept of neuroplasticity. The RT program consisted of eight exercises of the main muscle groups (Appendix 4 in SI). The exercises were performed with elastic bands and dumbbells. Initially, subjects were encouraged to start with minimum intensity (light elastic band and 1 kg dumbbells) and were instructed to progress when the following criteria were met: (a) exercise did not produce pain greater than 3-4/10 VAS, (b) no adverse response (increased pain or general discomfort) occurred within 48 h of program exercise, and (c) when (a) and (b) completed three consecutive days of training, progression in intensity was made. The established progression levels are detailed in SI (Appendix 5). In addition to the exercise program, the experimental group suspended pharmacological treatment for the duration of the treatment.

#### 2.5.2. Usual Care Group (AE + FE)

The AE program consisted of outdoor walking sessions, initially lasting 15–20 min and gradually progressing to 40–45 min. To facilitate the completion of the indicated program, heart rate (HR) was not monitored, as it has been shown not to be necessary to find the optimal intensity [30]. Instead, the rate of perceived exertion (RPE) was used, a reliable and widespread tool for assessing the subjective level of fatigue [31]. This scale takes values from 6 to 20 points, and a score of 12 (fairly light) to 13 (somewhat hard) has been found to be equivalent to moderate intensity (64%–76% of maximum HR) [32]. Thus, participants were instructed to start walking at a gentle pace and gradually increase to an RPE 12-13. In combination, an FE program used by Assumpçao et al. [33] was prescribed (Appendix 6 in SI). Three sets of 20 s were performed in the maintained position of each exercise. Subjects in the usual care group were allowed to continue with pharmacological treatment for FM symptoms, provided they were on medical prescription.

### 2.6. Statistical Analysis

IBM SPSS Statistics 26 was used for all statistical analyses. It was evaluated whether the analyzed variables conformed to normality using the Shapiro–Wilk test. In case of adjustment to normality, the repeated measures ANOVA test and mixed factorial ANOVA were used. Otherwise, Friedman's ANOVA test was used. The initial homogeneity was determined between the experimental and usual care groups of all demographic and clinical variables.

The values obtained in the “mean differences” and in the “percentages of changes” in the two groups were then compared. The effect size was estimated by calculating Cohen's *d* coefficient. In the case that such differences were not normal, we used the Mann–Whitney *U* test and the effect size was found by calculating Rosenthal's *r* with the formula: *r* = *Z*/√*N* [34]. An “intention-to-treat” analysis was performed in the study of the effects of the applied interventions. This study shows the preliminary results of a larger, long-term project, so the sample size was chosen for convenience to show the preliminary findings to the scientific community.

### 2.7. Minimum Clinically Important Difference (MCID)

The MCID of the primary outcomes was evaluated whenever possible. To determine the MCID for pain intensity, we followed the standards proposed by the Initiative on Methods, Measurement, and Pain Assessment in Clinical Trials (IMMPACT) [35], which established reference points to interpret clinically important changes in pain intensity on a numerical scale of 0 to 10 in clinical trials evaluating chronic pain. Consistent with this, the Philadelphia Panel [36] developed the relative benefit standard of 15% pain reduction, based on a broad analysis by experts in rheumatology and biostatistics. Overall, the reference values for the MCID were as follows: (a) a decrease of 10%–20% is considered *minimally important*; (b) a decrease of more than 30% is considered *moderately important*; and (c) a decrease of more than 50% is considered *substantially important*.

Regarding disability, Bennet et al. [37] showed that a 14% reduction in FIQ represented the MCID for people with fibromyalgia, so this value was taken as a reference. We have not found any studies that evaluated MCID in symptoms related to CS, so only the MCID of the variables pain intensity and disability was evaluated.

## 3. Results

### 3.1. Basic Characteristics of the Patients

**Figure 1:**
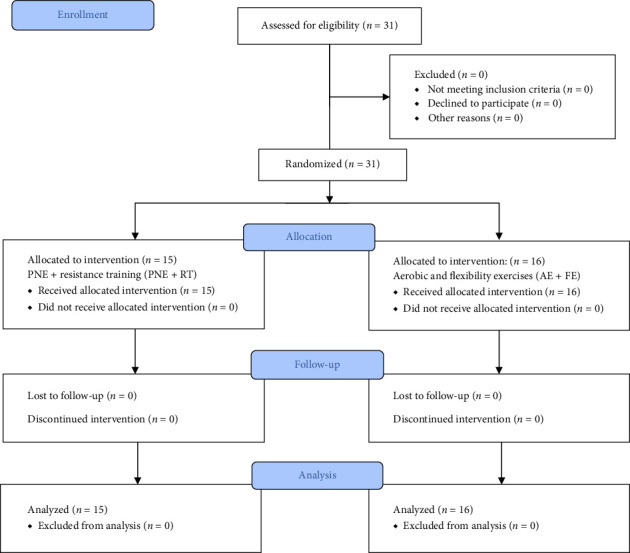
CONSORT flow diagram of study.

A total of 31 women (mean age 51.65 ± 8.82 years) were randomly divided into two groups: PNE + RT group (*n* = 15) and AE + FE group (*n* = 16). The flow diagram is presented in Figure 1.

Table 1 shows the values of the clinical and sociodemographic characteristics of the participants included in the study. No statistically significant differences were found between both groups. No adverse effects were reported.  Appendix 7 in SI shows the results of the normality tests.

### 3.2. Pain Intensity

Statistically significant improvements in pain intensity were observed only within the PNE + RT group (Table 2). These improvements were maintained across all follow-up points when compared to baseline (T0), see Appendix 8 in SI. Between-group comparisons also showed that the PNE + RT group achieved significantly greater reductions in pain intensity than the AE + FE group between T0 and T1, both in absolute difference and percentage of change, with a large effect size (*d* = 0.78 and *d* = 0.82, respectively) (Table 3).

Regarding MCID, the PNE + RT group showed clinically improvements in pain intensity at all follow-up points when compared to baseline: T0-T1 (25.79%), T0–T2 (21.94%), T0–T3 (32.09%), and T0–T4 (27.89%). Only the T0-T1 comparison reached statistical significance (*p*=0.03). In the AE + FE group, clinically relevant changes were observed at T0–T2 (15.29%) and T0–T3 (16.93%), but these differences were not statistically significant. In the between-group comparison, the PNE + RT group showed a clinically relevant improvement over the AE + FE group at T0-T1 (18.56%) (Table 3).

### 3.3. Disability

Both groups showed statistically significant within-group improvements in disability from baseline (T0) to all subsequent measurements (Appendix 9 in SI, and Table 2). However, no statistically significant between-group differences were observed at any time point (Table 3).

Regarding MCID, both interventions obtained clinically important improvements in disability when comparing T0 with each follow-up point. In the PNE + RT group, the percentage changes were 21.08% (T1), 21.10% (T2), 15.47% (T3), and 27.84% (T4). Similarly, the AE + FE group showed changes of 22.27%, 25.02%, 25.50%, and 27.20%, respectively. However, no clinically important differences were observed between the groups at any time point (Table 3).

### 3.4. Symptoms Related to CS

The PNE + RT group showed statistically significant within-group improvements in symptoms related to CS from baseline (T0) to all follow-up points (Appendix 10 in SI, and Table 4). However, no statistically significant between-group differences were observed at any time point.

### 3.5. MHS

Statistically significant within-group improvements were observed only in left-hand MHS in the PNE + RT group. No significant between-group differences were found for either hand at any time point. See Appendix 11 and 12 in SI, and Table 4.

### 3.6. PPT

The PNE + RT group showed statistically significant within-group improvements in the PPT of both trapezius muscles (Appendix 13 and 14 in SI, and Table 5). Between-group analysis also showed statistically significant differences in favor of the PNE + RT group, both in absolute change and percentage of change (Table 3).

The PPT of the quadriceps muscles also showed statistically significant within-group improvement in the PNE + RT group, both on the right (Appendix 15 in SI, and Table 4) and on the left (Appendix 16 in SI, and Table 5). However, no statistically significant between-group differences were observed at any time point (Tables 3 and 4).

### 3.7. Stiffness

No significant changes were observed in any of the stiffness-related parameters in the within-group comparisons (Appendix 17–23 in SI).

## 4. Discussion

To our knowledge, this is the first study to evaluate the effects of adding a PNE program to a RT intervention in women with fibromyalgia. The preliminary results suggest that this combined intervention (PNE + RT) appears to be more effective than the AE + FE program in improving short- and long-term pain intensity, as well as PPT in the trapezius muscle in the long term.

The PNE + RT program also led to improvements in disability, CS-related symptoms (short and long term), left-hand MHS, and PPT in the quadriceps muscle (long term), although it did not show statistically significant superiority over the AE + FE program in these outcomes. One possible explanation for the lack of between-group differences in some variables is that participants in the PNE + RT group discontinued their pharmacological treatment, which may have biased the results against this group.

The AE + FE program was only effective in improving disability (short and long term). Neither intervention significantly improved right-hand MHS or stiffness at the assessed points. Further research with larger sample sizes and better control of pharmacological variables is needed to confirm these findings.

We could justify our findings by considering that both pain intensity and PPT are measures of clinical symptoms, suggesting that subjective improvements do seem to be maintained long term after a PNE + RT program. With respect to stiffness, it is likely that a longer duration of exercise or higher intensity is required to produce structural changes. When using clinical criteria for progression, the intensity used was probably lower than necessary to produce adaptations in muscle architecture. Regarding the effects in MHS, all participants were right-handed; therefore, it appears that PNE + RT produced improvements only on the nondominant side. This could be because the first adaptations of RT are mainly explained by neural adaptations, which could contribute to normalizing strength asymmetries [38–40]. This fact, together with the improvement mainly in clinical symptoms, would support the idea that the benefits of PNE + RT could be due, at least in part, to neuroplastic changes.

The design of our intervention aimed to increase patient autonomy and improve self-efficacy, so we decided to use training progression criteria based on clinical symptoms and indications for self-management. To our knowledge, this is the first study to use such criteria in chronic pain populations. Some authors have already used pain monitoring (≤ 5/10 VAS and subsequent 24-h monitoring) during physical exercise, showing favorable results [41, 42]. However, this was performed in subjects with acute pain, where exercise-induced hypoalgesia (EIH) is not altered. Instead, we recommend using more conservative criteria for patients with chronic pain: (A) reporting pain levels below 3-4/10 on VAS, (B) monitoring symptoms for 48 hours, (C) meeting criteria A and B on three consecutive days of training.

Regarding MCID, the PNE + RT group outperformed MCID in pain intensity in both the short, medium, and long term; it is moderately significant (more than 30%) in the medium term (3 months), showing superiority over the AE + FE group in the short and medium term. Regarding disability, both groups showed clinically important improvements in all measurements performed (short, medium, and long term), although neither showed superiority over the other. These results suggest that both interventions may be useful when the objective is to improve the impact of the disease on quality of life. However, if the objective is to improve pain intensity, an intervention based on PNE + RT could be a better option.

We have not found any study that analyzes the effects of combining PNE with strength training in women with fibromyalgia. However, only one study has evaluated the effects of adding PNE to a therapeutic exercise program. Ceballos-Laita et al. [43]. analyzed the effects of a mixed exercise program with PNE compared to mixed exercise alone. These authors found that adding PNE to a therapeutic exercise program improved pain intensity in the short term, but not in the long term. However, they did not find significant improvements in disability, assessed with the FIQ, in any of the measurements performed. Our results, on the contrary, showed improvements in both the short and long term in both variables (pain intensity and disability). This controversy could be due to the characteristics of the interventions used. Strength training induces greater changes in the nervous system than other physical exercise modalities [38], which could contribute to a longer duration of clinical benefits. However, studies that have directly compared strength training with other therapeutic exercise modalities have found conflicting results.

There are several limitations to this study. First, the sample size was small, as expected in a pilot trial. Second, neither patients nor therapists were blinded. Although this is common in exercise-based interventions, it may influence the interpretation of outcomes.

Third, due to the number of variables assessed, some secondary outcomes were only measured at baseline (T0) and after the follow-up period (T4). As a result, we could not determine whether changes occurred immediately after the intervention. Further research should address this gap.

Fourth, pharmacological treatment was discontinued in the experimental group (PNE + RT). This may have introduced a bias against that group. Future studies should maintain consistent medication use between groups to allow clearer comparisons.

Lastly, the lack of significant changes in MHS and stiffness could be related to the small sample size. These findings should be viewed as preliminary and may guide future studies exploring specific or extended interventions.

## 5. Conclusions

The findings of this study suggest that the combination of PNE and RT is more effective than an AE + FE program in improving pain intensity in the short term and PPT in the trapezius muscle in the long term. PNE + RT is also effective in improving disability, CS-related symptoms (short and long term), pain intensity, left MHS, and PPT in the quadriceps muscle (long-term), although it is not more effective than AE + FE. The AE + FE program is only effective in improving disability. More RCTs are needed to corroborate these findings.

## Figures and Tables

**Table 1:** Clinical and demographic characteristics of patients.

Variable	Total (*n* = 31)	AE + FE (*n* = 16)	PNE + RT (*n* = 15)	*p* value
Age (years)	51.65 ± 8.82	53.94 ± 6.74	49.20 ± 10.28	*p*=0.137^a^
Weight (kg)	70.19 ± 14.29	68.63 ± 9.05	71.87 ± 18.54	*p*=0.547^b^
Height (m)	1.64 ± 0.05	1.65 ± 0.04	1.64 ± 0.05	*p*=0.575^a^
BMI (kg/m^2^)	26.08 ± 5.36	24.82 (22.32; 26.67)	25.39 (22.57; 28.95)	*p*=0.599^c^
Duration of symptoms (years)	12.29 ± 8.32	10.00 (8.50; 14.00)	10.00 (6.50; 12.00)	*p*=0.572^c^
Marital status (%)				
Married	19 (59.4%)	9 (56.3%)	10 (66.7%)	*p*=0.765^d^
Single	5 (15.6%)	2 (12.5%)	3 (20.0%)	
Divorced/separated	5 (15.6%)	4 (25.0%)	1 (6.7%)	
Widowed	2 (6.3%)	1 (6.3%)	1 (6.7%)	
Educational level (%)				
High school graduate or less	16 (51.61%)	11 (68.8%)	5 (33.3%)	*p*=0.158^d^
Some college/trade school	8 (25.81%)	3 (18.8%)	5 (33.3%)	
College degree or graduate degree	7 (22.58%)	2 (12.5%)	5 (33.3%)	
Smoker (%)				
No	25 (78.1%)	13 (81.3%)	3 (18.8%)	*p*=0.999^e^
Yes	6 (18.1%)	12 (80.0%)	3 (20.0%)	
Manual dominance, *n* (%)				
Right	31 (100%)	16 (100%)	15 (100%)	*p*=0.999^e^
Left	0 (0%)	0 (0%)	0 (0%)	
Medication, *n* (%)				
No	9 (29.1%)	4 (25.0%)	5 (33.3%)	*p*=0.223^d^
NSAID	7 (21.9%)	3 (18.8%)	4 (26.7%)	
Opioids	6 (18.8%)	4 (25.0%)	2 (13.3%)	
Benzodiazepines	4 (12.5%)	4 (25.0%)	0 (0.0%)	
Antidepressants	2 (6.3%)	0 (0.0%)	2 (13.3%)	
Antiepileptics	3 (9.4%)	1 (6.3%)	2 (13.3%)	
VAS pretest (0–100)	79.68 ± 15.38	80.00 (70.00; 100.00)	80.00 (70.00; 90.00)	*p*=0.520^c^
FIQ pretest (0–100)	77.34 ± 17.79	81.14 (70.16; 93.52)	82.29 (67.48; 87.53)	*p*=0.770^c^
CSI pretest (0–100)	71.65 ± 14.98	69.88 ± 10.81	73.53 ± 18.66	*p*=0.515^b^
Right MHS pretest (kg)	18.77 ± 5.85	18.75 ± 18.80	18.80 ± 6.22	*p*=0.982^a^
Left MHS pretest (kg)	14.65 ± 7.41	15.25 ± 6.99	14.00 ± 8.04	*p*=0.647^a^
Pressure pain threshold (kg)				
Trapezius muscle, right	1.73 ± 0.82	1.82 ± 0.65	1.63 ± 0.98	*p*=0.518^a^
Trapezius muscle, left	1.81 ± 0.75	1.81 ± 0.72	1.82 ± 0.81	*p*=0.957^a^
Quadriceps muscle, right	3.55 ± 1.73	3.30 ± 1.24	3.81 ± 2.15	*p*=0.419^a^
Quadriceps muscle, left	3.56 ± 1.59	3.29 ± 0.98	3.86 ± 2.06	*p*=0.344^b^
Stiffness (N/m)				
Trapezius muscle, right	312.81 ± 51.19	330.50 (279.50; 353.50)	269.00 (260.50; 346.00)	*p*=0.338^c^
Trapezius muscle, left	324.42 ± 51.93	331.94 ± 37.01	316.40 ± 64.64	*p*=0.414^a^
Quadriceps muscle, right	297.06 ± 43.82	298.50 ± 41.55	295.53 ± 47.54	*p*=0.854^a^
Quadriceps muscle, left	299.19 ± 57.21	302.44 ± 56.51	295.73 ± 59.72	*p*=0.750^a^

*Note:* Values are expressed as mean ± SD (standard deviation), median (Q1; Q3), or percentages; AE + FE: aerobic and flexibility exercises; PNE + RT: a combination of pain neuroscience education and resistance training. *p* > 0.05 indicates that there were no differences between the intervention groups.

Abbreviations: BMI = body mass index, CSI = central sensitization inventory, FIQ = fibromyalgia impact questionnaire, MHS = maximum handgrip strength, NSAID = nonsteroidal anti-inflammatory drugs, and VAS = visual analog scale.

^a^ *t*-Student test.

^b^ *t*-Welch test.

^c^Mann–Whitney *U* test.

^d^Pearson's exact test.

^e^Fisher's exact test.

**Table 2:** Within-group analysis of the variables pain intensity and disability.

Variable	Intervention group	Measurements		Overall	Within-group analysis (*p* value)			
					T0 vs T1	T0 vs T2	T0 vs T3	T0 vs T4
EVA (0–100)	AE + FE	T0	80.00 (70.00; 100.00)	*p*=0.064 (Friedman's ANOVA)	*p*=0.580	*p*=0.060	*p*=0.130	*p*=0.240
		T1	75.00 (70.00; 90.00)					
		T2	70.00 (60.00; 80.00)					
		T3	70.00 (50.00; 80.00)					
		T4	80.00 (60.00; 80.00)					
	PNE + RT	T0	76.67 ± 15.88	*p* < 0.001^∗^ (*η*_*p*_^2^ = 0.318)	*p* < 0.001^∗^	*p*=0.021^∗^	*p*=0.001^∗^	*p*=0.002^∗^
		T1	58 ± 21.11					
		T2	60.67 ± 26.58					
		T3	53.33 ± 25.54					
		T4	56.00 ± 23.54					

FIQ (0–100)	AE + FE	T0	79.11 ± 15.46	*p* < 0.001^∗^ (*η*_*p*_^2^ = 0.377)	*p* < 0.001^∗^ (*η*_*p*_^2^ = 0.468)	*p* < 0.001^∗^ (*η*_*p*_^2^ = 0.538)	*p* < 0.001^∗^ (*η*_*p*_^2^ = 0.513)	*p* < 0.001^∗^ (*η*_*p*_^2^ = 0.489)
		T1	61.03 ± 20.79					
		T2	59.66 ± 21.11					
		T3	61.36 ± 21.03					
		T4	58.09 ± 23.63					
	PNE + RT	T0	82.29 (67.48; 87.53)	*p*=0.017^∗^ Friedman's ANOVA	*p*=0.005^∗^	*p*=0.015^∗^	*p*=0.018^∗^	*p*=0.002^∗^
		T1	60.71 (50.91; 75.74)					
		T2	66.00 (45.65; 72.05)					
		T3	71.43 (40.15; 74.45)					
		T4	64.43 (28.45; 74.79)					

*Note:* Values are expressed as mean ± SD or median (Q1; Q3). AE + FE: aerobic and flexibility exercises; PNE + RT: a combination of pain neuroscience education and resistance training; *η*_*p*_^2^: partial eta squared coefficient; T0: preintervention; T1: postintervention; T2: 1 month follow-up; T3: 3 months follow-up; T4: 6 months follow-up.

Abbreviations: FIQ = fibromyalgia impact questionnaire; VAS = visual analog scale.

^∗^ *p* < 0.05, statistically significant differences.

**Table 3:** Analysis between groups of change in the variables: intensity of pain, disability, and pressure pain threshold (left and right upper trapezius muscles and left quadriceps).

Variable			Differences between measurements		Between-group analysis of change	
			AE + FE	PNE + RT		
					Intergroup difference (95% CI)	Significance effect size
VAS (0–100)	T0 vs T1	Difference	−6.94 ± 14.07	−18.67 ± 15.98	11.73 (0.69, 22.77)	*p*=0.038^a^/*d* = 0.78^∗^
		% change	−7.23 ± 17.58	−25.79 ± 26.89	18.56 (1.98, 35.14)	*p*=0.030^a^/*d* = 0.82^∗^
	T0 vs T2	Difference	−13.13 ± 15.37	−16.00 ± 23.84	2.88 (−11.76, 17.51)	*p*=0.691^a^/*d* = 0.14
		% change	−15.29 ± 18.30	−21.94 ± 35.43	6.65 (−13.87, 27.17)	*p*=0.513^a^/*d* = 0.24
	T0 vs T3	Difference	−14.38 ± 20.65	−23.33 ± 21.27	8.96 (−6.44, 24.36)	*p*=0.244^a^/*d* = 0.43
		% change	−16.93 ± 26.84	−32.09 ± 32.69	15.16 (−6.75, 37.07)	*p*=0.168^a^/*d* = 0.51
	T0 vs T4	Difference	−11.88 ± 17.97	−20.67 ± 20.86	8.79 (−5.48, 23.06)	*p*=0.218^a^/*d* = 0.46
		% change	−13.74 ± 25.03	−27.89 ± 31.35	14.15 (−6.62, 34.92)	*p*=0.174^a^/*d* = 0.50

FIQ (0–100)	T0 vs T1	Difference	−18.11 ± 18.11	−15.56 ± 18.96	−2.5 (6.66, −16.14)	*p*=0.708^a^/*d* = 0.14
		% change	−22.27 ± 24.28	−21.08 ± 23.61	−1.19 ± 8.60 (−18.80, 16.42)	*p*=0.891^a^/*d* = 0.05
	T0 vs T2	Difference	−29.09 (−30.22; −6.59)	−10.95 (−25.87; −2.69)	—	*p*=0.304^b^/*r* = 0.18
		% change	−25.02 ± 24.05	−21.10 ± 20.85	−3.91 (−20.50, 12.66)	*p*=0.632^a^/*d* = 0.17
	T0 vs T3	Difference	−17.76 ± 14.68	−15.05 ± 18.29	−2.70 (5.94, −14.85)	*p*=0.653^a^/*d* = 0.12
		% change	−25.50 (−30.66; −13.26)	−15.47 (−40.43; −1.52)	—	*p*=0.553^b^/*r* = 0.11
	T0 vs T4	Difference	−17.14 (−31.99; −5.25)	−12.98 (−26.22; −3.88)	—	*p*=0.580^b^/*r* = 0.10
		% change	−27.20 ± 24.98	−27.84 ± 28.95	0.65 (−19.18, 20.47)	*p*=0.947^a^/*d* = 0.02

Pressure pain threshold (kg) (T0 vs T4)	Trapezius, right	Difference	0.30 (−0.15; 0.70)	1.40 (0.55; 2.15)	—	*p*=0.006^b^/*r* = 0.49^∗^
		% change	14.33 (−12.94; 59.82)	100.00 (24.87; 173; 21)	—	*p*=0.007^b^/*r* = 0.48^∗^
	Trapezius, left	Difference	0.25 (−0.05; 0.70)	1.10 (0.75; 1.65)	—	*p*=0.002^b^/*r* = 0.56^∗^
		% change	17.69 (−1.92; 43.33)	64.71 (45.70; 99.84)	—	*p*=0.003^b^/*r* = 0.54^∗^
	Quad, left	Difference	0.79 ± 1.82	1.66 ± 1.90		*p*=0.205^a^/*d* = 0.01
		% change	4.08 (−1.56; 10.58)	13.18 (6.04; 16.42)	—	*p*=0.058/*r* = 0.34

*Note:* Values are expressed as mean ± SD or median (Q1; Q3). AE + FE: aerobic and flexibility exercises; PNE + RT: a combination of pain neuroscience education and resistance training; *d*: Cohen's *d*; *r*: Rosenthal's *r*; T0: preintervention; T1: postintervention; T2: 1 month follow-up; T3: 3 months follow-up; T4: 6 months follow-up; quad: quadriceps muscle.

Abbreviations: CI = confidence interval, FIQ = fibromyalgia impact questionnaire, and VAS = visual analog scale.

^a^ *t*-Student test.

^b^Mann–Whitney *U* test.

^∗^ *p* < 0.05, statistically significant differences.

**Table 4:** Results of the mixed factor analysis of the variables symptoms related to central sensitization, maximum handgrip strength (right and left), and pressure pain threshold (left quadriceps muscle).

**Variable**					

SCI	Time × treatment interaction	*F*_(4, 116)_ = 2.61, *p*=0.057; *η*_*p*_^2^: = 0.083			
	Between-group analysis	*F*_(1, 29)_ = 0.36, *p*=0.554; *η*_*p*_^2^ = 0.012			
	Intergroup difference (95% CI)	Time	AE + FE	PNE + RT	MD/*p* value
		T1	65.31 (12.89)	55.47 (23.82)	9.85 (−4.10; 23.79) *p*=0.159*d* = 0.52
		T2	61.94 (11.98)	61.73 (20.77)	0.20 (−12.15; 12.56) *p*=0.973*d* = 0.01
		T3	66.81 (13.08)	62.07 (21.01)	4.75 (−8.02; 17.51) *p*=0.453*d* = −0.27
		T4	63.25 (12.63)	57.87 (22.44)	5.38 (−7.88; 18.65) *p*=0.413*d* = −0.30
	Within-group analysis	*F*_(4, 116)_ = 8.65, *p* ≤ 0.001; *η*_*p*_^2^ = 0.230^∗^			
	Intragroup difference (95% CI)	AE + FE	T0 vs T1	4.56 (−7.86; 16.99); *p*=0.999	
			T0 vs T2	7.94 (−0.73; 16.61); *p*=0.094	
			T0 vs T3	3.06 (−6.37; 12.50); *p*=0.999	
			T0 vs T4	6.63 (−4.72; 17.97); *p*=0.865	
		PNE + RT	T0 vs T1	18.07 (5.24; 30.90); *p*=0.002^∗^	
			T0 vs T2	11.80 (2.85; 20.75); *p*=0.004^∗^	
			T0 vs T3	11.47 (1.72; 21.21); *p*=0.012^∗^	
			T0 vs T4	15.67 (3.95; 27.38); *p*=0.003^∗^	

Maximum handgrip strength, right (kg)	Time × treatment interaction	*F*_(1, 29)_ = 1.03, *p*=0.319; *η*_*p*_^2^ = 0.034			
	Between-group analysis	*F*_(1, 29)_ = 0.38, *p*=0.543; *η*_*p*_^2^ = 0.013			
	Intergroup difference (95% CI)	Time	AE + FE	PNE + RT	MD/*p* value
		T4	18.38 ± 6.48	20.67 ± 6.08	−2.29 (−6.92; 2.33) *p*=0.319*d* = 0.36
	Within-group analysis	*F*_(1, 29)_ = 0.46, *p*=0.505; *η*_*p*_^2^ = 0.015			
	Intragroup difference (95% CI)	AE + FE	0.38 (−2.77; 3.52) *p*=0.809		
		PNE + RT	−1.87 (−5.12; 1.38), *p*=0.249		

Maximum handgrip strength, left (kg)	Time × treatment interaction	*F*_(1, 29)_ = 2.60, *p*=0.118; *η*_*p*_^2^ = 0.082			
	Between-group analysis	*F*_(1, 29)_ = 0.02, *p*=0.895; *η*_*p*_^2^ = 0.001			
	Intergroup difference (95% CI)	Time	AE + FE	PNE + RT	MD/*p* value
		T4	17.50 ± 7.28	19.40 ± 7.03	−1.90 (−7.16; 3.36) *p*=0.466*d* = 0.27
	Within-group analysis	*F*_(1, 29)_ = 15.30, *p*=0.001; *η*_*p*_^2^ = 0.345^∗^			
	Intragroup difference (95% CI)	AE + FE	−2.25 (−5.03; 0.53); *p*=0.109		
		PNE + RT	−5.40 (−8.27; −2.53), *p*=0.001^∗^		

Pressure pain threshold (kg) quadriceps, right	Time × treatment interaction	*F*_(1, 29)_ = 2.39, *p*=0.133; *η*_*p*_^2^ = 0.076			
	Between-group analysis	*F*_(1, 29)_= 3.07, *p*=0.090; *η*_*p*_^2^ = 0.096			
	Intergroup difference (95% CI)	Time	Control	Exp	MD/*p* value
		T4	3.92 ± 2.03	5.79 ± 2.95	−1.80 (−3.65; 0.06) *p*=0.06*d* = 0.74
	Within-group analysis	*F*_(1, 29)_ = 9.25, *p*=0.005; *η*_*p*_^2^ = 0.242^∗^			
	Intragroup difference (95% CI)	AE + FE	−0.62 (−1.80; 0.56), *p*=0.292		
		PNE + RT	−1.90 (−3.12; −0.68); *p*=0.003^∗^		

*Note:* Values are expressed as mean ± SD or median (Q1; Q3). AE + FE: aerobic and flexibility exercises; PNE + RT: a combination of pain neuroscience education and resistance training; T0: preintervention; T1: postintervention; T2: 1 month follow-up; T3: 3 months follow-up; T4: 6 months follow-up. *η*_*p*_^2^: partial eta squared coefficient; *d*: Cohen's *d*.

Abbreviations: CI = confidence interval, CSI = central sensitization inventory, and MD = mean difference.

^∗^ *p* < 0.05, statistically significant differences.

**Table 5:** Within‐group analysis of the variable pressure pain threshold. Right and left upper trapezius muscles, and left quadriceps.

Variable		Group	Measurements		Within-group analysis (*p* value)
			T0	T4	*p* value
Pressure pain threshold (kg)	Trapezius, right	AE + FE	1.80 (1.35; 2.35)	1.95 (1.55; 2.80)	*p*=0.116^a^
		PNE + RT	1.50 (1.05; 1.70)	2.90 (2.00; 4.25)	*p*=0.001^a∗^
	Trapezius, left	AE + FE	1.81 ± 0.72	2.19 ± 1.20	*p*=0.119^b^
		PNE + RT	1.61 (1.35; 2.15)	2.90 (2.20; 4.45)	*p*=0.001^a∗^
	Quadriceps, left	AE + FE	3.45 (2.75; 4.00)	3.65 (3.15; 4.50)	*p*=0.127^a^
		PNE + RT	3.86 ± 2.06	5.51 ± 2.78	*p*=0.004^b∗^

*Note:* Values are expressed as mean ± SD or median (Q1; Q3). AE + FE: aerobic and flexibility exercises; PNE + RT: a combination of pain neuroscience education and resistance training; T0: preintervention; T4: 6 months follow-up. Right and left upper trapezius muscles, and left quadriceps.

^a^Wilcoxon signed-rank test.

^b^ *t*-Student test.

^∗^ *p* < 0.05, statistically significant differences.

## Data Availability

The data that support the findings of this study are openly available in Research Repository University of Seville at https://doi.org/10.12795/11441/162394, reference number 11441/162394.

## Supporting Information

Appendix 1: The figure shows the position and procedure used to assess the pain threshold to pressure in the upper trapezius and quadriceps muscles using the electronic pressure algometer (Wagner FPXTM). Appendix 2: The figure shows the position and procedure used to assess of maximum handgrip strength using the hydraulic hand dynamometer (Jamar). Appendix 3: The figure shows the position and procedure used to assess stiffness using Myoton Pro. Appendix 4: The table shows the resistance training program realized by the experimental group. Appendix 5: The table shows the proposed progression levels for resistance training program realized by the experimental group. Appendix 6: The table shows the flexibility training program realized by the usual care group. Appendix 7: The figure shows the results obtained in the two intervention groups for pain intensity. Appendix 8: The figure shows the results obtained in the two intervention groups for disability. Appendix 9: The figure shows the results obtained in the two intervention groups for symptoms related to central sensitization. Appendix 10: The figure shows the results obtained in the two intervention groups for left maximum handgrip strength. Appendix 11: The figure shows the results obtained in the two intervention groups for right maximum handgrip strength. Appendix 12: The figure shows the results obtained in the two intervention groups for the pressure pain threshold on the right upper trapezius muscle. Appendix 13: The figure shows the results obtained in the two intervention groups for the pain threshold to pressure on the left upper trapezius muscle. Appendix 14: The figure shows the results obtained in the two intervention groups for the pressure pain threshold on the right quadriceps muscle. Appendix 15: The figure shows the results obtained in the two intervention groups for the pressure pain threshold on the left quadriceps muscle. Appendix 16: The figure shows the results obtained in the two intervention groups for stiffness on the right upper trapezius muscle. Appendix 17: The figure shows the results obtained in the two intervention groups for stiffness on the left upper trapezius muscle. Appendix 18: The figure shows the results obtained in the two intervention groups for stiffness on the right quadriceps muscle. Appendix 19: The figure shows the results obtained in the two intervention groups for stiffness on the left quadriceps muscle. Appendix 20: The table shows the observed change in right upper trapezius muscle stiffness within each intervention group (within-group). Appendix 21: The table shows the observed change in the stiffness of the right upper trapezius muscle between the two intervention groups (between-group). Appendix 22: The table shows the results obtained, both intra and intergroup, in the stiffness of the left upper trapezius muscles and the right and left quadriceps muscles. Appendix 23: The table shows the results obtained, both intra and intergroup, in the stiffness of the left upper trapezius muscles and the right and left quadriceps muscles. Appendix 24: CONSORT 2010 checklist used for reporting this randomized trial.
