# Supplementary material for: Pain Neuroscience Education and Resistance Training in Women With Fibromyalgia: A Randomized Control Pilot Study
Source: Pain Res Manag. 2025 Jul 10;2025:7550108. doi: 10.1155/prm/7550108 (PMC12271695; doi:10.1155/prm/7550108)
Supplement: Supporting Information — Additional supporting information can be found online in the Supporting Information section. [file 7550108.f1.docx]

**Supplementary Material**

| **Supplemental Materials** | **Page** |
| --- | --- |
| Appendix 1. The figure shows the position and procedure used to assess the pain threshold to pressure in the upper trapezius and quadriceps muscles using the electronic pressure algometer (Wagner FPXTM) | 2 |
| Appendix 2. The figure shows the position and procedure used to assess of maximum handgrip strength using the hydraulic hand dynamometer (Jamar). | 2 |
| Appendix 3. The figure shows the position and procedure used to assess stiffness using Myoton Pro®. | 2 |
| Appendix 4. The table shows the resistance training program realized by the experimental group. | 3 |
| Appendix 5. The table shows the proposed progression levels for resistance training program realized by the experimental group. | 3 |
| Appendix 6. The table shows the flexibility training program realized by the active control group. | 4 |
| Appendix 7. The table shows the results of the normality tests. | 5 |
| Appendix 8. The figure shows the results obtained in the two intervention groups for pain intensity. | 7 |
| Appendix 9. The figure shows the results obtained in the two intervention groups for disability. | 7 |
| Appendix 10. The figure shows the results obtained in the two intervention groups for symptoms related to central sensitization. | 8 |
| Appendix 11. The figure shows the results obtained in the two intervention groups for left maximum handgrip strength. | 8 |
| Appendix 12. The figure shows the results obtained in the two intervention groups for right maximum handgrip strength. | 9 |
| Appendix 13. The figure shows the results obtained in the two intervention groups for the pressure pain threshold on the right upper trapezius muscle. | 9 |
| Appendix 14. The figure shows the results obtained in the two intervention groups for the pain threshold to pressure on the left upper trapezius muscle. | 10 |
| Appendix 15. The figure shows the results obtained in the two intervention groups for the pressure pain threshold on the right quadriceps muscle | 10 |
| Appendix 16. The figure shows the results obtained in the two intervention groups for the pressure pain threshold on the left quadriceps muscle. | 11 |
| Appendix 17. The figure shows the results obtained in the two intervention groups for stiffness on the right upper trapezius muscle. | 11 |
| Appendix 18. The figure shows the results obtained in the two intervention groups for stiffness on the left upper trapezius muscle. | 12 |
| Appendix 19. The figure shows the results obtained in the two intervention groups for stiffness on the right quadriceps muscle. | 12 |
| Appendix 20. The figure shows the results obtained in the two intervention groups for stiffness on the left quadriceps muscle. | 13 |
| Appendix 21. The table shows the observed change in right upper trapezius muscle stiffness within each intervention group (within-group). | 13 |
| Appendix 22. The table shows the observed change in the stiffness of the right upper trapezius muscle between the two intervention groups (between-group). | 13 |
| Appendix 23. The table shows the results obtained, both intra and intergroup, in the stiffness of the left upper trapezius muscles and the right and left quadriceps muscles. | 14 |
| Appendix 24. CONSORT 2010 checklist used for reporting this randomized trial. | 15 |
| REFERENCES | 17 |

**Appendix. 1.** Assessment of pressure pain threshold in the upper trapezius and quadriceps muscles


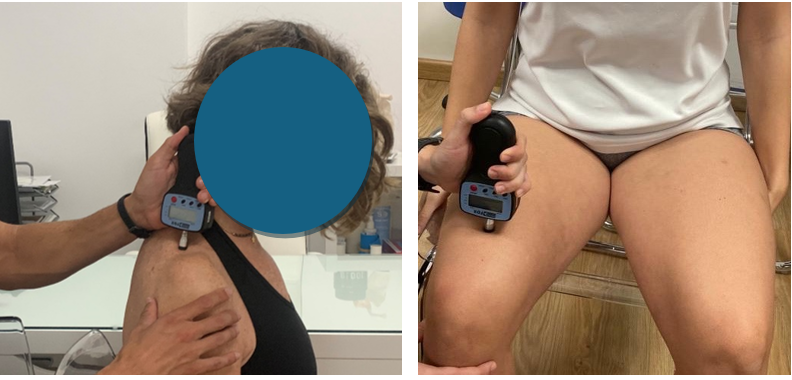

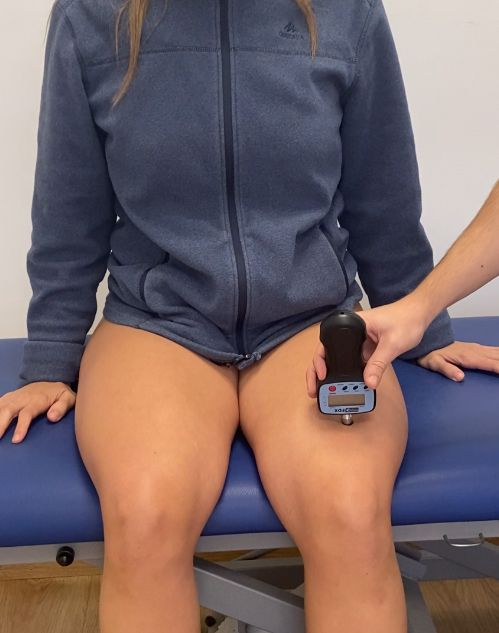


**Appendix 2.** Assessment of maximum handgrip strength.


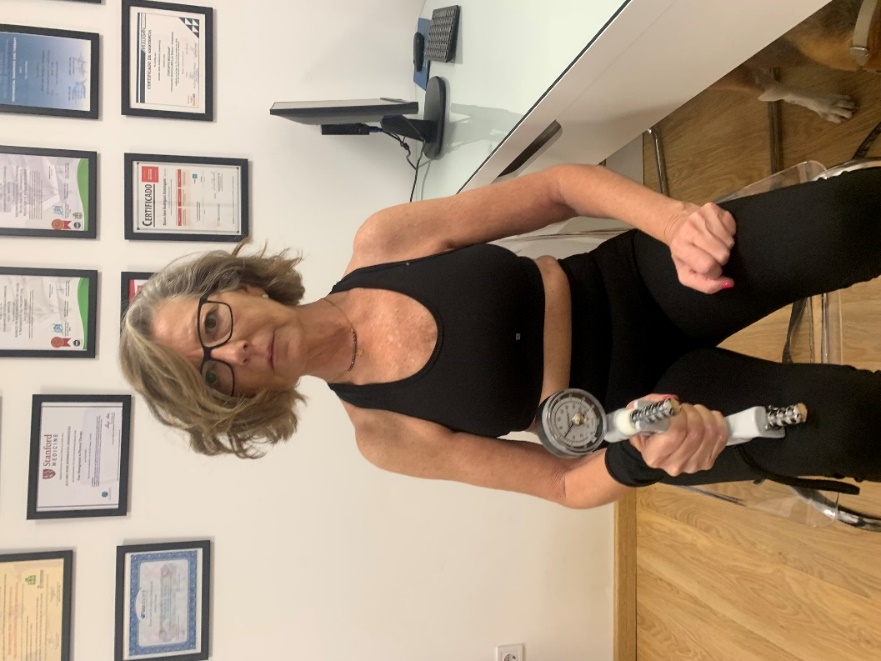


**Appendix 3.** Assessment of stiffness in the upper trapezius and quadriceps muscles

**
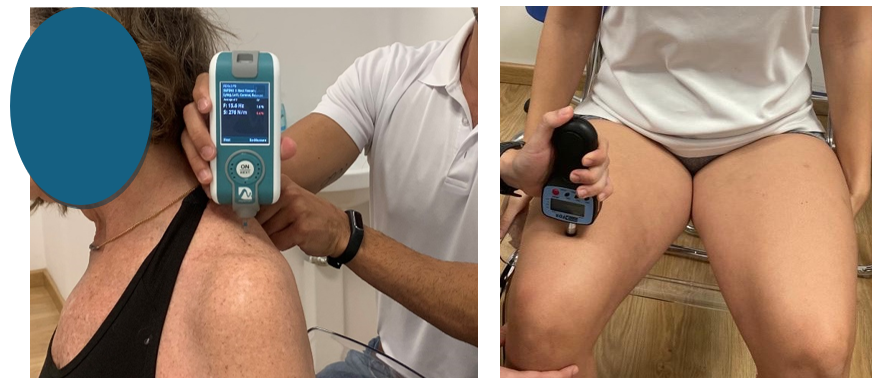

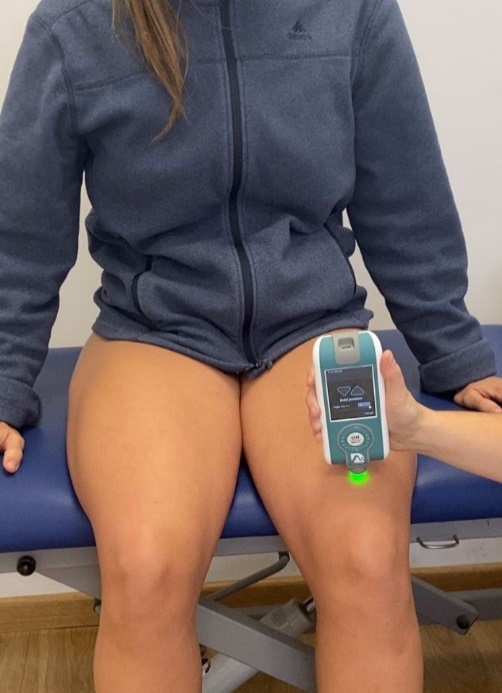
**

**Appendix 4.** Resistance training program

| 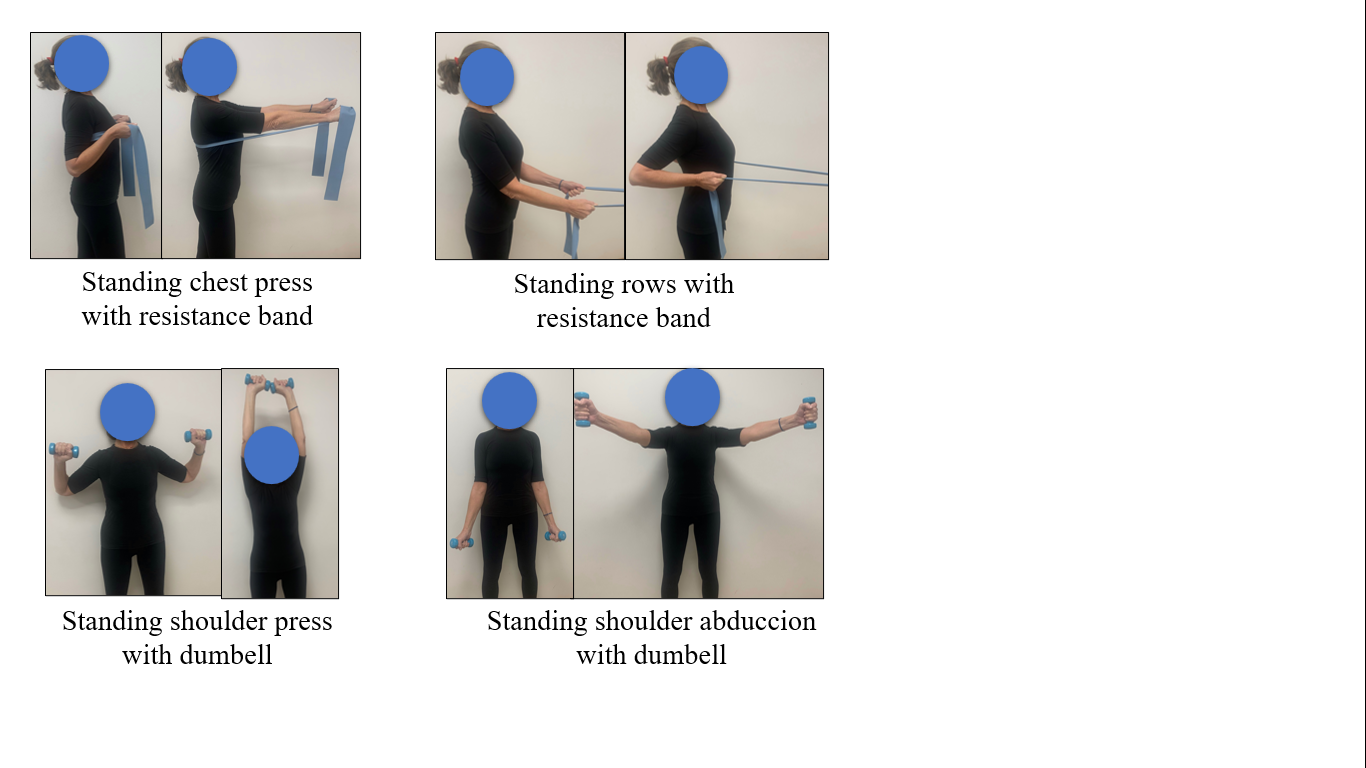 |
| --- |
| 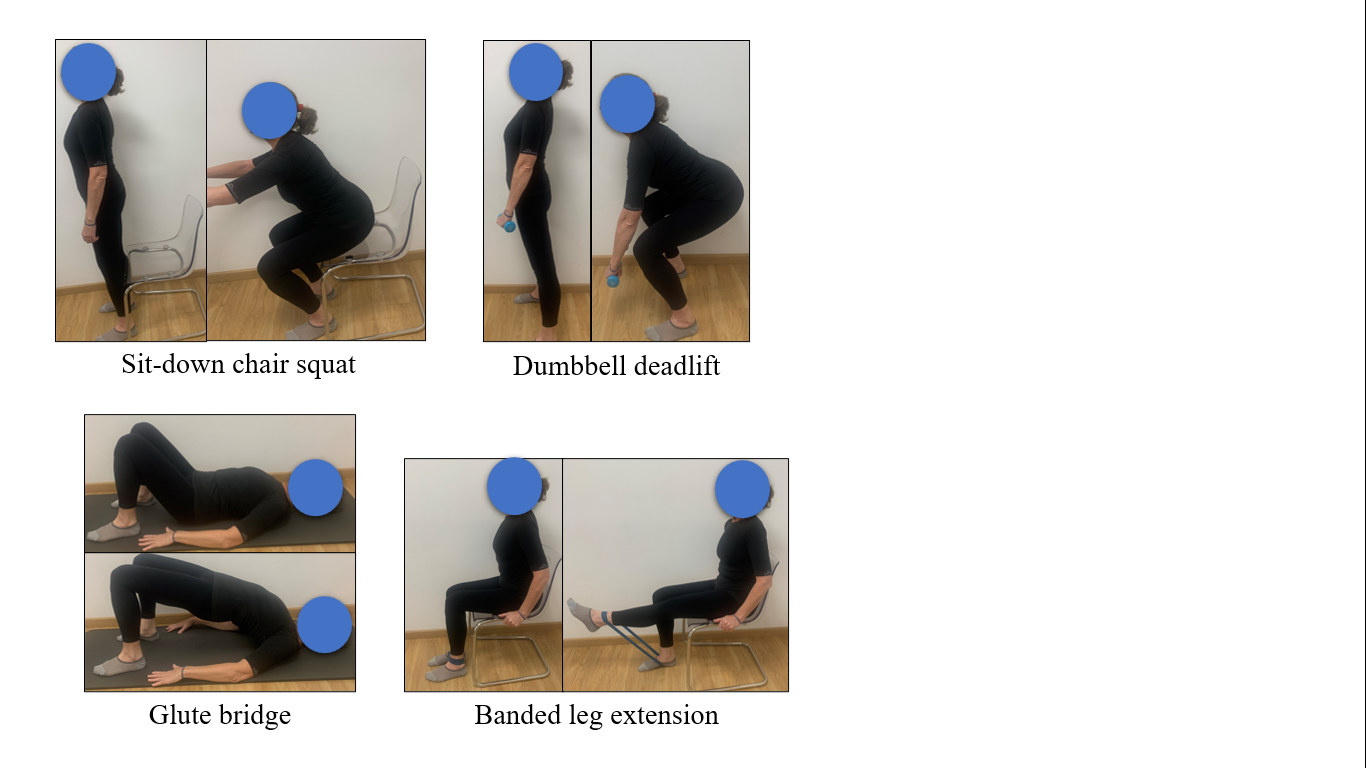 |

**Appendix 5.** Progression levels for resistance training program.

| Level | Intensity in the upper extremity (dumbbell and elastic band) | Intensity in the upper extremity (dumbbell and elastic band) | Sets | Repetitions | Total volume of repetitions per exercise |
| --- | --- | --- | --- | --- | --- |
| Level 1 | 1 kg / easy | 2 kg / easy | 1 | 15-20 | 15-20 |
| Level 2 | 1.5 kg / low | 4 kg / low | 2 | 12-15 | 24-30 |
| Level 3 | 2 kg / moderate | 6 / moderate | 2 | 15-18 | 30-36 |
| Level 4 | 2.5 kg / hard | 8 kg / hard | 3 | 10-12 | 30-45 |
| Level 5 | 3 kg / maximal | 10 kg / maximal | 3 | 12-15 | 36-45 |

Intensity progression with elastic bands was performed according to Colado et al.[1]

**Appendix 6**. Flexibility exercises program,

| 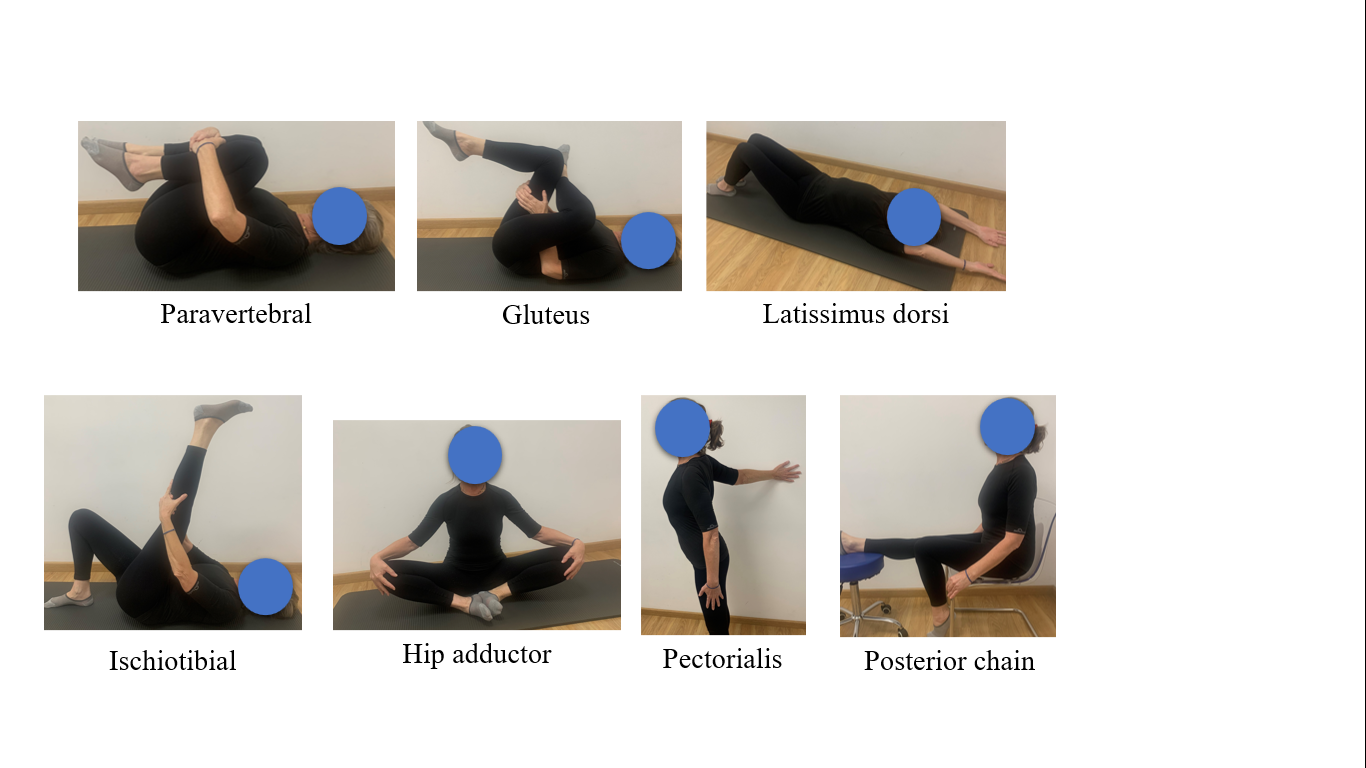 |
| --- |

This program was proposed by Assumpçao et al.[2]

**Appendix 7**. Results of the normality tests.

| **Normality test** | | | | | | | |
| --- | --- | --- | --- | --- | --- | --- | --- |
|  | Treatment | Kolmogorov-Smirnov^a^ | | | Shapiro-Wilk | | |
|  |  | Statistic | df | Sig. | Statistic | df | Sig. |
| Age, years | FE+AE | .132 | 16 | .200* | .951 | 16 | .507 |
|  | RT+PNE | .129 | 15 | .200* | .961 | 15 | .701 |
| Weight, kg | FE+AE | .153 | 16 | .200* | .950 | 16 | .483 |
|  | RT+PNE | .207 | 15 | .084 | .885 | 15 | .056 |
| Height, cm | FE+AE | .149 | 16 | .200* | .945 | 16 | .420 |
|  | RT+PNE | .182 | 15 | .193 | .953 | 15 | .569 |
| Body Mass Index | FE+AE | .230 | 16 | .024 | .862 | 16 | .021 |
|  | RT+PNE | .182 | 15 | .195 | .887 | 15 | .059 |
| Duration since diagnosis, years | FE+AE | .229 | 16 | .025 | .767 | 16 | .001 |
|  | RT+PNE | .311 | 15 | <.001 | .782 | 15 | .002 |
| VAS pre-test | FE+AE | .256 | 16 | .006 | .801 | 16 | .003 |
|  | RT+PNE | .204 | 15 | .093 | .902 | 15 | .103 |
| VAS final intervention | FE+AE | .165 | 16 | .200* | .932 | 16 | .260 |
|  | RT+PNE | .219 | 15 | .051 | .887 | 15 | .061 |
| VAS post-1 month | FE+AE | .173 | 16 | .200* | .936 | 16 | .301 |
|  | RT+PNE | .223 | 15 | .043 | .920 | 15 | .194 |
| VAS post-3 month | FE+AE | .220 | 16 | .037 | .928 | 16 | .229 |
|  | RT+PNE | .270 | 15 | .004 | .903 | 15 | .107 |
| VAS post-6 month | FE+AE | .239 | 16 | .015 | .872 | 16 | .029 |
|  | RT+PNE | .167 | 15 | .200* | .941 | 15 | .394 |
| FIQ pre-test | FE+AE | .116 | 16 | .200* | .939 | 16 | .342 |
|  | RT+PNE | .202 | 15 | .099 | .835 | 15 | .011 |
| FIQ final intervention | FE+AE | .121 | 16 | .200* | .938 | 16 | .331 |
|  | RT+PNE | .155 | 15 | .200* | .925 | 15 | .228 |
| FIQ post-1 month | FE+AE | .114 | 16 | .200* | .969 | 16 | .821 |
|  | RT+PNE | .154 | 15 | .200* | .975 | 15 | .920 |
| FIQ post-3 month | FE+AE | .112 | 16 | .200* | .973 | 16 | .880 |
|  | RT+PNE | .211 | 15 | .072 | .923 | 15 | .211 |
| FIQ post-6 month | FE+AE | .142 | 16 | .200* | .932 | 16 | .263 |
|  | RT+PNE | .216 | 15 | .059 | .930 | 15 | .273 |
| CSI pre-test | FE+AE | .201 | 16 | .084 | .920 | 16 | .169 |
|  | RT+PNE | .122 | 15 | .200* | .930 | 15 | .274 |
| CSI final intervention | FE+AE | .115 | 16 | .200* | .986 | 16 | .995 |
|  | RT+PNE | .142 | 15 | .200* | .934 | 15 | .313 |
| CSI post-1 month | FE+AE | .244 | 16 | .012 | .924 | 16 | .197 |
|  | RT+PNE | .109 | 15 | .200* | .947 | 15 | .476 |
| CSI post-3 month | FE+AE | .137 | 16 | .200* | .961 | 16 | .679 |
|  | RT+PNE | .120 | 15 | .200* | .971 | 15 | .866 |
| CSI post-6 month | FE+AE | .107 | 16 | .200* | .982 | 16 | .978 |
|  | RT+PNE | .119 | 15 | .200* | .977 | 15 | .943 |
| **(continuation of previous table)**   \| **Normality test** \| \| \| \| \| \| \| \| \| --- \| --- \| --- \| --- \| --- \| --- \| --- \| --- \| \|  \| Treatment \| Kolmogorov-Smirnov^a^ \| \| \| Shapiro-Wilk \| \| \| \|  \| Statistic \| df \| Sig. \| Statistic \| df \| Sig. \| \| Right grip Strength pre-test \| FE+AE \| .216 \| 16 \| .044 \| .943 \| 16 \| .384 \| \| RT+PNE \| .182 \| 15 \| .193 \| .899 \| 15 \| .092 \| \| Right MHS post-6 month \| FE+AE \| .224 \| 16 \| .031 \| .898 \| 16 \| .074 \| \| RT+PNE \| .192 \| 15 \| .142 \| .930 \| 15 \| .270 \| \| Left MHS pre-test \| FE+AE \| .142 \| 16 \| .200* \| .980 \| 16 \| .963 \| \| RT+PNE \| .157 \| 15 \| .200* \| .937 \| 15 \| .342 \| \| Left grip Strength post-6 month \| FE+AE \| .116 \| 16 \| .200* \| .960 \| 16 \| .654 \| \| RT+PNE \| .211 \| 15 \| .072 \| .938 \| 15 \| .359 \| \| PPT right trapezius pre-test \| FE+AE \| .176 \| 16 \| .200 \| .935 \| 16 \| .292 \| \| RT+PNE \| .243 \| 15 \| .017 \| .885 \| 15 \| .056 \| \| PPT right trapezius post-6 month \| FE+AE \| .371 \| 16 \| .000 \| .460 \| 16 \| .000 \| \| RT+PNE \| .204 \| 15 \| .094 \| .770 \| 15 \| .002 \| \| PPT left trapezius pre-test \| FE+AE \| .166 \| 16 \| .200* \| .947 \| 16 \| .437 \| \| RT+PNE \| .244 \| 15 \| .017 \| .912 \| 15 \| .143 \| \| PPT left trapezius post-6 month \| FE+AE \| .210 \| 16 \| .057 \| .911 \| 16 \| .121 \| \| RT+PNE \| .244 \| 15 \| .016 \| .819 \| 15 \| .007 \| \| PPT right quadriceps pre-test \| FE+AE \| .104 \| 16 \| .200* \| .977 \| 16 \| .936 \| \| RT+PNE \| .146 \| 15 \| .200* \| .946 \| 15 \| .461 \| \| PPT right quadriceps post-6 month \| FE+AE \| .200 \| 16 \| .087 \| .913 \| 16 \| .129 \| \| RT+PNE \| .193 \| 15 \| .138 \| .928 \| 15 \| .253 \| \| PPT left quadriceps pre-test \| FE+AE \| .233 \| 16 \| .020 \| .903 \| 16 \| .091 \| \| RT+PNE \| .129 \| 15 \| .200* \| .933 \| 15 \| .298 \| \| PPT left quadriceps post-6 month \| FE+AE \| .226 \| 16 \| .028 \| .849 \| 16 \| .013 \| \| RT+PNE \| .198 \| 15 \| .117 \| .927 \| 15 \| .250 \| \| Stiffness right trapezius pre-test \| FE+AE \| .159 \| 16 \| .200* \| .963 \| 16 \| .710 \| \| RT+PNE \| .284 \| 15 \| .002 \| .847 \| 15 \| .016 \| \| Stiffness right trapezius poRT-6 month \| FE+AE \| .149 \| 16 \| .200* \| .928 \| 16 \| .228 \| \| RT+PNE \| .125 \| 15 \| .200* \| .952 \| 15 \| .557 \| \| Stiffness left trapezius pre-test \| FE+AE \| .178 \| 16 \| .190 \| .927 \| 16 \| .219 \| \| RT+PNE \| .137 \| 15 \| .200* \| .891 \| 15 \| .069 \| \| Stiffness left trapezius post-6 month \| FE+AE \| .115 \| 16 \| .200* \| .965 \| 16 \| .751 \| \| RT+PNE \| .201 \| 15 \| .107 \| .921 \| 15 \| .202 \| \| Stiffness right quad pre-test \| FE+AE \| .169 \| 16 \| .200* \| .954 \| 16 \| .555 \| \| RT+PNE \| .126 \| 15 \| .200* \| .956 \| 15 \| .622 \| \| Stiffness right quad post-6 month \| FE+AE \| .109 \| 16 \| .200* \| .977 \| 16 \| .938 \| \| RT+PNE \| .126 \| 15 \| .200* \| .972 \| 15 \| .880 \| \| Stiffness left quad pre-test \| FE+AE \| .207 \| 16 \| .066 \| .896 \| 16 \| .069 \| \| RT+PNE \| .098 \| 15 \| .200* \| .971 \| 15 \| .869 \| \| Stiffness left quad post-6 month \| FE+AE \| .122 \| 16 \| .200* \| .964 \| 16 \| .740 \| \| RT+PNE \| .138 \| 15 \| .200* \| .949 \| 15 \| .510 \| \| Sig: significance; df:degree freedom; AE+FE: aerobic and flexibility exercises; PNE+RT: a combination of pain neuroscience education and resistance training; kg: kilograms; cm: centimeters; VAS: visual analogic scale; FIQ: fibromyalgia impact questionnaire; CSI: central sensitization inventory; MHS: maximum handgrip strength; PPT: pressure pain threshold  a. Correction of Lilliefors' significance. *This is a lower limit of true significance. \| \| \| \| \| \| \| \| | | | | | | | |

**Appendix 8.** Results of the five measurements of pain intensity (measured with the VAS scale from 0 to 100 points) in the two treatment groups.


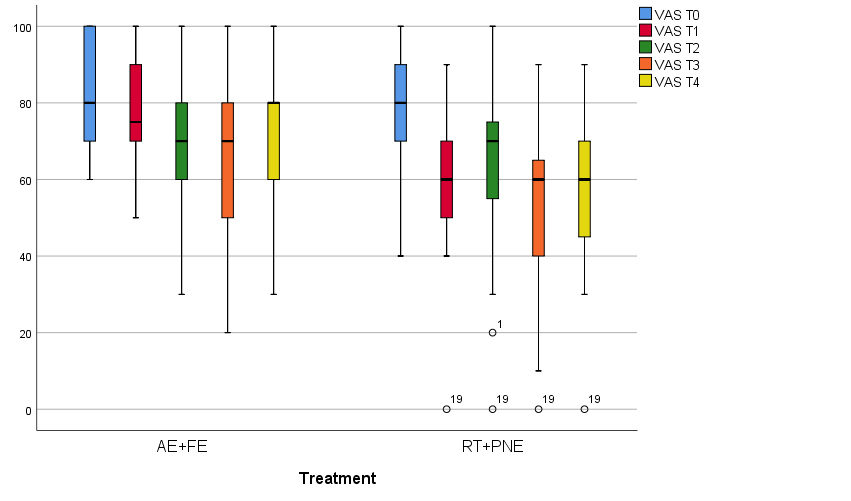


VAS: visual analogic scale; AE+FE: aerobic and flexibility exercises; PNE+RT: a combination of pain neuroscience education and resistance training. T0: pre-intervention; T1: post-intervention; T2: one month follow-up; T3: three months follow-up; T4: six months follow-up.

**Appendix 9.** Results of the five measurements of disability (measured with the FIQ) in the two treatment groups.


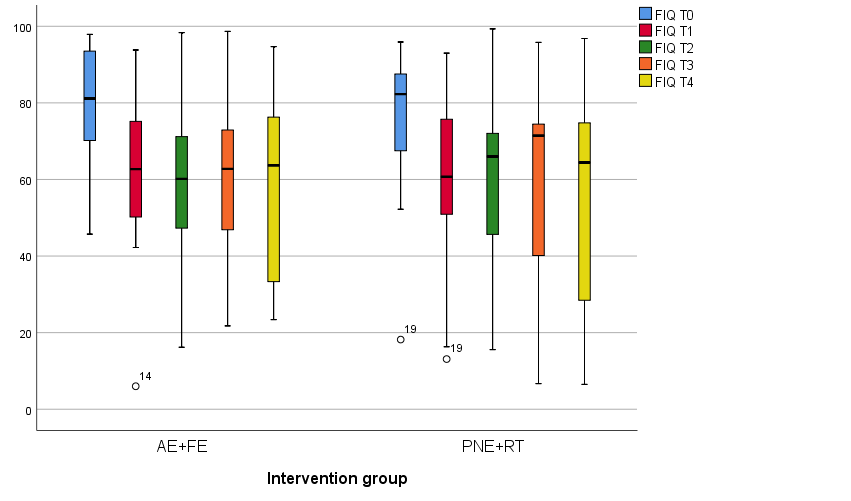


FIQ: fibromyalgia impact questionnaire; AE+FE: aerobic and flexibility exercises; PNE+RT: a combination of pain neuroscience education and resistance training. T0: pre-intervention; T1: post-intervention; T2: one month follow-up; T3: three months follow-up; T4: six months follow-up.

**Appendix 10**. Results of the five measurements of symptoms related to central sensitization (measured with the CSI) in the two treatment groups.


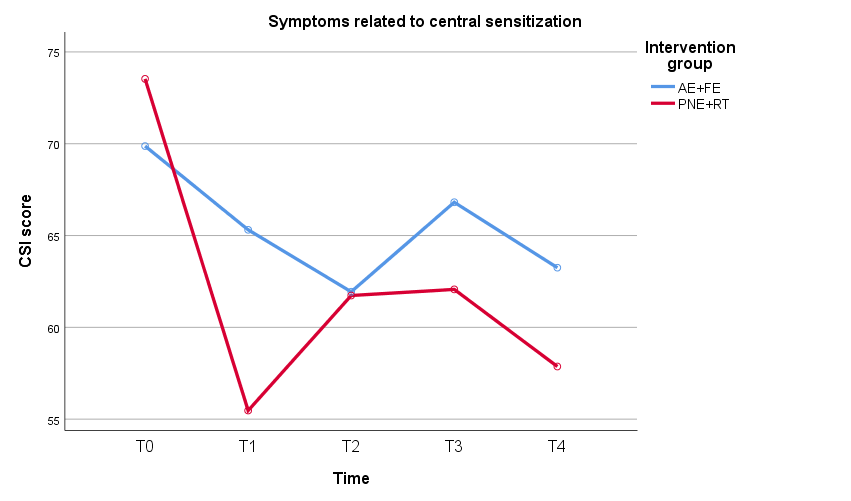


CSI: central sensitization inventory; AE+FE: aerobic and flexibility exercises; PNE+RT: a combination of pain neuroscience education and resistance training. T0: pre-intervention; T1: post-intervention; T2: one month follow-up; T3: three months follow-up; T4: six months follow-up.

**Appendix 11**. Results of maximum handgrip strength (measured with dynamometer) in the two treatment groups. Left hand.


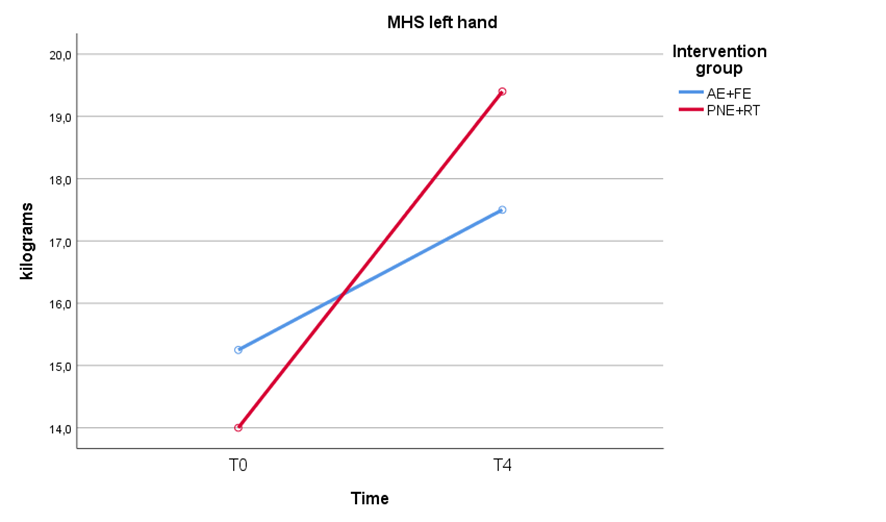


MHS: maximum handgrip strength; AE+FE: aerobic and flexibility exercises; PNE+RT: a combination of pain neuroscience education and resistance training. T0: pre-intervention; T4: six months follow-up.

**Appendix 12**. Results of maximum handgrip strength (measured with dynamometer) in the two treatment groups. Right hand.


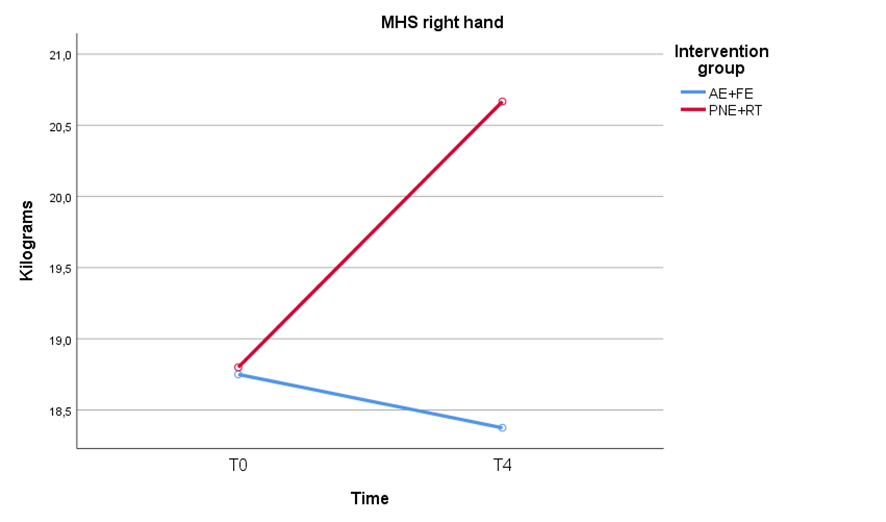


MHS: maximum handgrip strength; AE+FE: aerobic and flexibility exercises; PNE+RT: a combination of pain neuroscience education and resistance training. T0: pre-intervention; T4: six months follow-up.

**Appendix 13**. Results of pressure pain threshold (measured with algometer) in the two treatment groups. Right upper trapezius muscle.


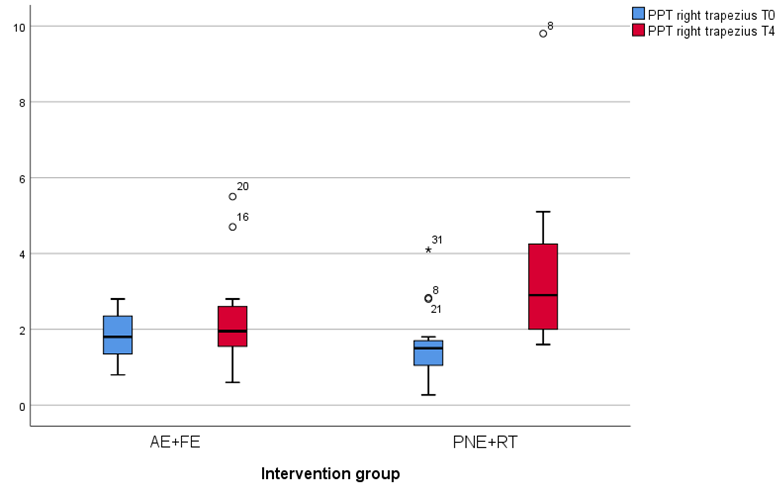


PPT: pressure pain threshold; AE+FE: aerobic and flexibility exercises; PNE+RT: a combination of pain neuroscience education and resistance training. T0: pre-intervention; T4: six months follow-up.

**Appendix 14**. Results of pressure pain threshold (measured with algometer) in the two treatment groups. Left upper trapezius muscle.


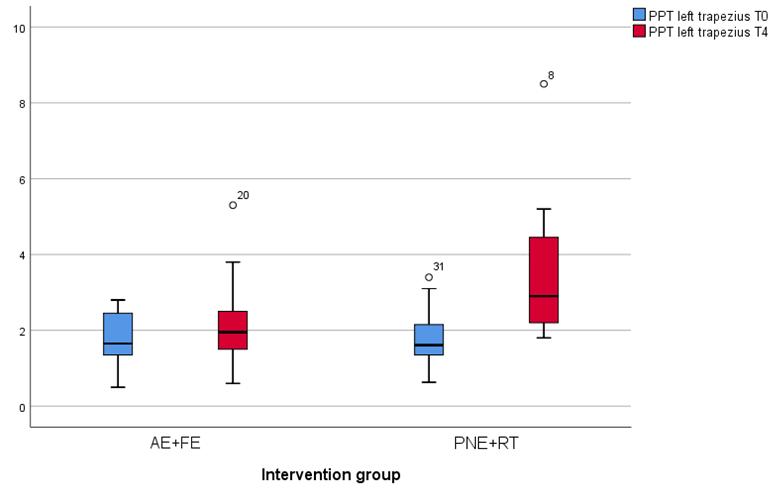


PPT: pressure pain threshold; AE+FE: aerobic and flexibility exercises; PNE+RT: a combination of pain neuroscience education and resistance training. T0: pre-intervention; T4: six months follow-up.

**Appendix 15**. Results of pressure pain threshold (measured with algometer) in the two treatment groups. Right quadriceps muscle.


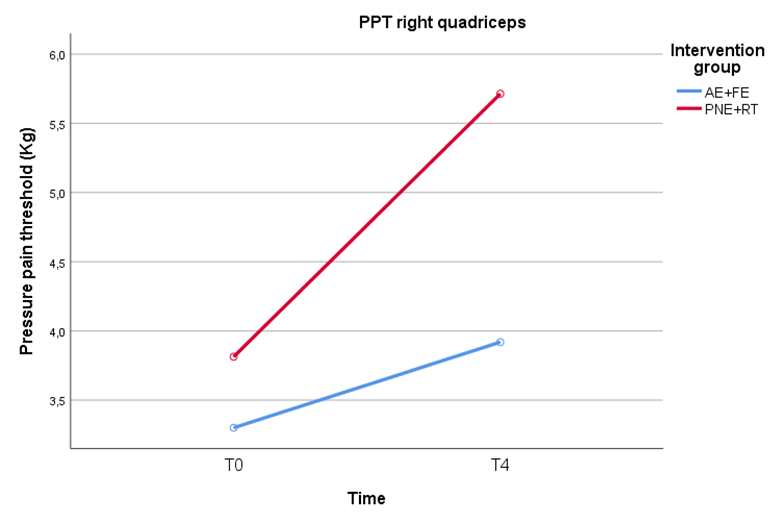


PPT: pressure pain threshold; AE+FE: aerobic and flexibility exercises; PNE+RT: a combination of pain neuroscience education and resistance training. T0: pre-intervention; T4: six months follow-up.

**Appendix 16**. Results of pressure pain threshold (measured with algometer) in the two treatment groups. Left quadriceps muscle.


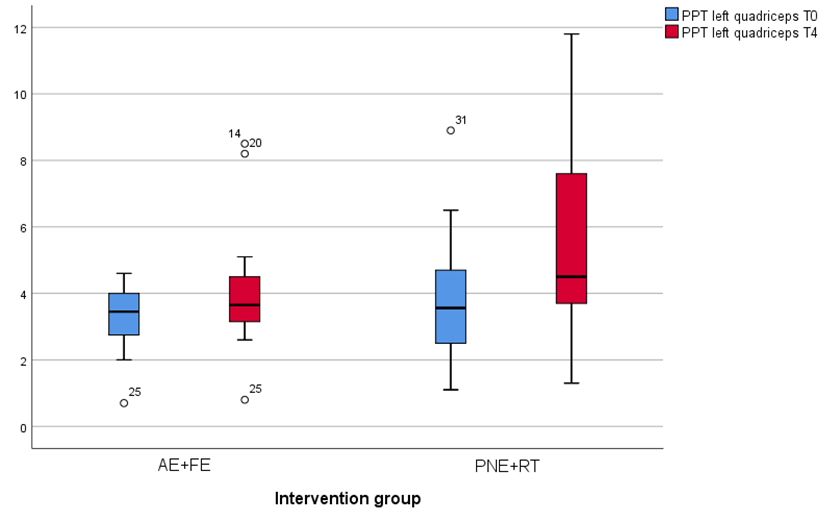


PPT: pressure pain threshold; AE+FE: aerobic and flexibility exercises; PNE+RT: a combination of pain neuroscience education and resistance training. T0: pre-intervention; T4: six months follow-up.

**Appendix 17**. Results of stiffness (measured with myotonometer) in the two treatment groups. Right upper trapezius muscle.


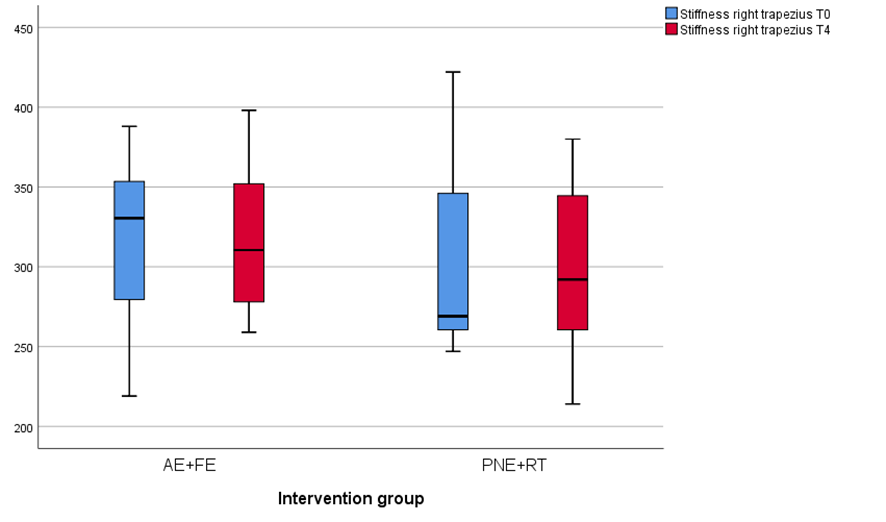


AE+FE: aerobic and flexibility exercises; PNE+RT: a combination of pain neuroscience education and resistance training. T0: pre-intervention; T4: six months follow-up.

**Appendix 18.** Results of stiffness (measured with myotonometer) in the two treatment groups. Left upper trapezius muscle.


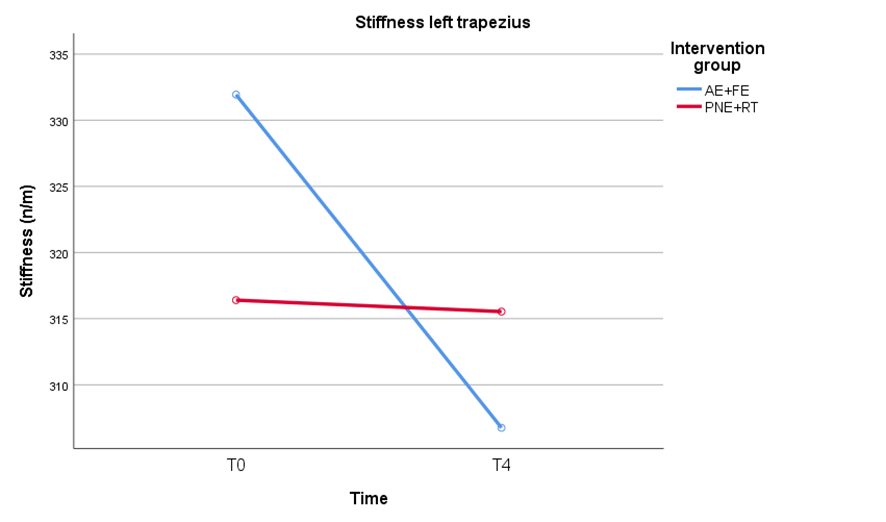


AE+FE: aerobic and flexibility exercises; PNE+RT: a combination of pain neuroscience education and resistance training. T0: pre-intervention; T4: six months follow-up.

**Appendix 19**. Results of stiffness (measured with myotonometer) in the two treatment groups. Right quadriceps muscle.


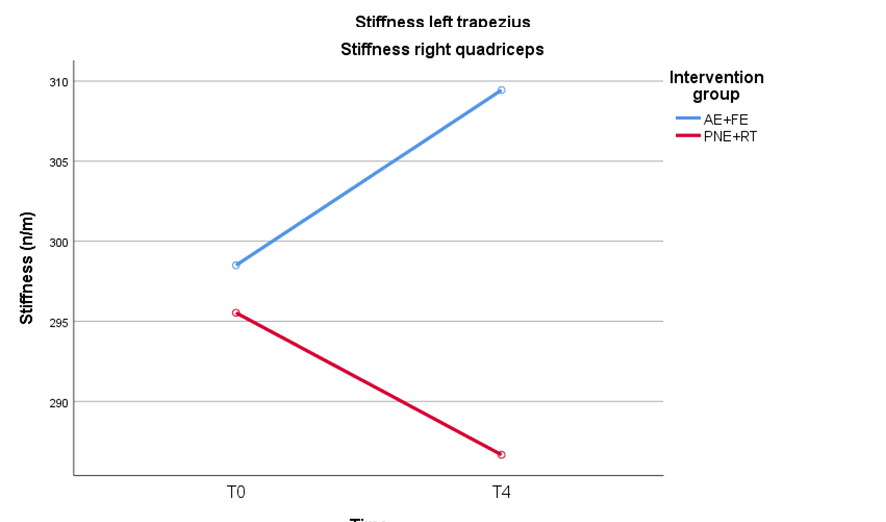


AE+FE: aerobic and flexibility exercises; PNE+RT: a combination of pain neuroscience education and resistance training. T0: pre-intervention; T4: six months follow-up.

**Appendix 20**. Results of stiffness (measured with myotonometer) in the two treatment groups. Left quadriceps muscle.


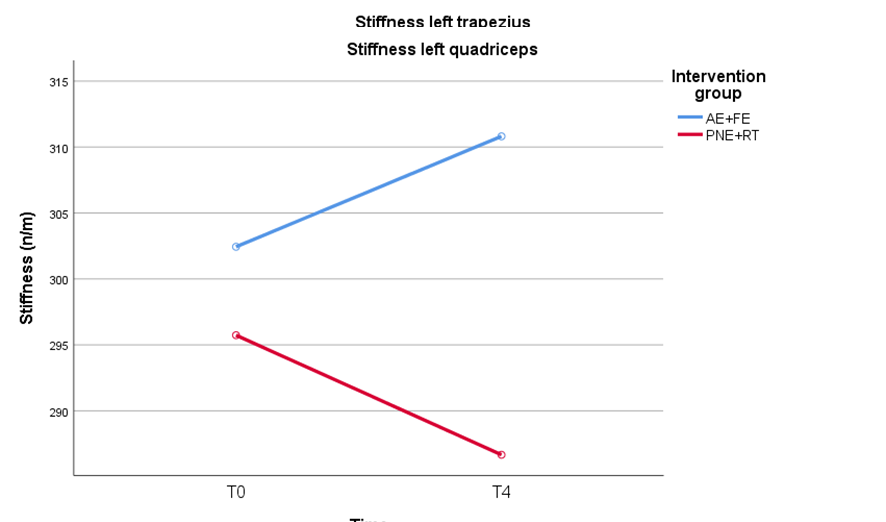


AE+FE: aerobic and flexibility exercises; PNE+RT: a combination of pain neuroscience education and resistance training. T0: pre-intervention; T4: six months follow-up.

.

**Appendix 21.** Within-group analysis of the stiffness. Right upper trapezius muscle.

| **Variable** | | **Group** | **Measurements** | | **Within-group analysis (p-value)** |
| --- | --- | --- | --- | --- | --- |
|  |  |  | **T0** | **T4** | **p-value** |
| **Stiffness (N/m)** | **Trapezius, right** | AE+FE | 317.69 ± 46.35 | 317.00 ± 44.81 | p =0.969^b^ |
|  |  | PNE+RT | 269 (260.50; 346.00) | 292.00 (260.50; 344.50) | p =0.268^a^ |

Values are mean ± SD or median (Q1; Q3). AE+FE: aerobic and flexibility exercises; PNE+RT: a combination of pain neuroscience education and resistance training. T0: pre-intervention; T4: six months follow-up.

^a^ Wilcoxon signed rank test; ^b^ t-Student test

**Appendix 22.** Between-group analysis of change in the stiffness. Right upper trapezius muscle.

| **Variable** | | | **Differences between measurements** | | **Between-group analysis of change** |
| --- | --- | --- | --- | --- | --- |
|  |  |  | **AE+FE** | **PNE+RT** |  |
|  |  |  |  |  | **Significance**  **Effect size** |
| **Stiffness (N/m)** | **Trapezius, right** | Difference | 00.69 ± 70.11 | 8.73 ± 41.69 | p =0.703^a^  d =0.14 |
|  |  | % change | -2.78 (-14.78; 21.76) | 4.52 (-6.12; 9.12) | p =0.813  r =0.04 |

Values are mean ± SD or median (Q1; Q3). AE+FE: aerobic and flexibility exercises; PNE+RT: a combination of pain neuroscience education and resistance training; d: Cohen´s d; r: Rosenthal´s r.

^a^ t-Student test; ^b^ Mann-Whitney U test

**Appendix 23.** Results of the mixed factor analysis of the variable stiffness. Right and left quadriceps muscles and left upper trapezius.

| Variable | |  | | | | | |
| --- | --- | --- | --- | --- | --- | --- | --- |
| **Stiffness (N/m)** | **Trapezius, left** | Time x treatment interaction | F_(1,29)_ = 1.144, p = 0.294; η^2^_p_ = 0.038 | | | | |
|  |  | Between-group analysis | F_(1,29)_ = 0.062, p = 0.805; η^2^_p_ = 0.002 | | | | |
|  |  | Intergroup difference  (95% CI) | Time | AE+FE | | PNE+RT | MD / p-value |
|  |  |  | T4 | 306.75 ± 50.23 | | 315.53 ± 41.05 | 15.54 (-22.84; 53.91) p = 0.414 d = 0.19 |
|  |  | Within-group analysis | F_(1,29)_ = 1.313, p = 0.261; η^2^_p_ = 0.043 | | | | |
|  |  | Intragroup difference  (95% CI) | AE+FE | | 25.188 (-7.16; 57.54)  p = 0.122 | | |
|  |  |  | PNE+RT | | 0.87 (-32.54; 34.28)  p = 0.958 | | |
|  | **Quadriceps, right** | Time x treatment interaction | F_(1,29)_ = 0.565, p = 0.458; η^2^_p_ = 0.019 | | | | |
|  |  | Between-group analysis | F_(1,29)_ = 0.928, p = 0.343; η^2^_p_ = 0.031 | | | | |
|  |  | Intergroup difference  (95% CI) | Time | AE+FE | | PNE+RT | MD / p-value |
|  |  |  | T4 | 309.44 ± 62.18 | | 286.67 ± 55.13 | 22.77 (-20.51; 66.05) p = 0.291 d = 0.39 |
|  |  | Within-group analysis | F_(1,29)_ = 0.006, p = 0.938; η^2^_p_ <0.001 | | | | |
|  |  | Intragroup difference  (95% CI) | AE+FE | | -10.937 (-48.42; 26.54)  p = 0.555 | | |
|  |  |  | PNE+RT | | 8.867 (-29.84; 47.58)  p = 0.643 | | |
|  | **Quadriceps, left** | Time x treatment interaction | F_(1,29)_ = 0.330, p = 0.570; η^2^_p_ = 0.011 | | | | |
|  |  | Between-group analysis | F_(1,29)_ = 0.996, p = 0.326; η^2^_p_ = 0.033 | | | | |
|  |  | Intergroup difference  (95% CI) | Time | AE+FE | | PNE+RT | MD / p-value |
|  |  |  | T4 | 310.81 ± 63.15 | | 286.67 ± 61.64 | 24.15 (-21.74; 70.03) p = 0.291 d = 0.39 |
|  |  | Within-group analysis | F_(1,29)_ = 0.001, p = 0.982; η^2^_p_ <0.001 | | | | |
|  |  | Intragroup difference  (95% CI) | AE+FE | | -8.375 (-51.60; 34.85)  p = 0.695 | | |
|  |  |  | PNE+RT | | 9.067 (-35.58; 53.71)  p = 0.681 | | |

Values are mean ± SD or median (Q1; Q3). CI: confidence interval; MD: mean difference; AE+FE: aerobic and flexibility exercises; PNE+RT: a combination of pain neuroscience education and resistance training; T0: pre-intervention; T4: six months follow-up.

η^2^_p_: partial Eta squared coefficient; d: Cohen´s d.

**Appendix 24.** CONSORT 2010 checklist used for reporting this randomized trial.

|  | Section/topic | No | CONSORT 2025 checklist item description | Reported on page no. |
| --- | --- | --- | --- | --- |
|  | **Title and abstract** | | |  |
|  | Title and structured abstract | 1a | Identification as a randomised trial | Tittle page |
|  |  | 1b | Structured summary of the trial design, methods, results, and conclusions | 4 |
|  | **Open science** | | |  |
|  | Trial registration | 2 | Name of trial registry, identifying number (with URL) and date of registration | Tittle page |
|  | Protocol and statistical analysis plan | 3 | Where the trial protocol and statistical analysis plan can be accessed | 7 |
|  | Data sharing | 4 | Where and how the individual de-identified participant data (including data dictionary), statistical code and any other materials can be accessed | Tittle page |
|  | Funding and conflicts of interest | 5a | Sources of funding and other support (eg, supply of drugs), and role of funders in the design, conduct, analysis and reporting of the trial | Tittle page |
|  |  | 5b | Financial and other conflicts of interest of the manuscript authors | Tittle page |
|  | **Introduction** | | | **6-7** |
|  | Background and rationale | 6 | Scientific background and rationale |  |
|  | Objectives | 7 | Specific objectives related to benefits and harms | 7 |
|  | **Methods** | | |  |
|  | Patient and public involvement | 8 | Details of patient or public involvement in the design, conduct and reporting of the trial | 7-8 |
|  | Trial design | 9 | Description of trial design including type of trial (eg, parallel group, crossover), allocation ratio, and framework (eg, superiority, equivalence, non-inferiority, exploratory) | 7-8 |
|  | Changes to trial protocol | 10 | Important changes to the trial after it commenced including any outcomes or analyses that were not prespecified, with reason | 8-9 |
|  | Trial setting | 11 | Settings (eg, community, hospital) and locations (eg, countries, sites) where the trial was conducted | 7 |
|  | Eligibility criteria | 12a | Eligibility criteria for participants | 8 |
|  |  | 12b | If applicable, eligibility criteria for sites and for individuals delivering the interventions (eg, surgeons, physiotherapists) | 8 |
|  | Intervention and comparator | 13 | Intervention and comparator with sufficient details to allow replication. If relevant, where additional materials describing the intervention and comparator (eg, intervention manual) can be accessed | 11-12 |
|  | Outcomes | 14 | Prespecified primary and secondary outcomes, including the specific measurement variable (eg, systolic blood pressure), analysis metric (eg, change from baseline, final value, time to event), method of aggregation (eg, median, proportion), and time point for each outcome | 9-11 |
|  | Harms | 15 | How harms were defined and assessed (eg, systematically, non-systematically) | 8-9 |
|  | Sample size | 16a | How sample size was determined, including all assumptions supporting the sample size calculation | 13 |
|  |  | 16b | Explanation of any interim analyses and stopping guidelines | 11-13 |
|  | Randomisation: |  |  | 8-11 |
|  | Sequence generation | 17a | Who generated the random allocation sequence and the method used |  |
|  |  | 17b | Type of randomisation and details of any restriction (eg, stratification, blocking and block size) | 8-11 |
|  |  |  |  | **Reported on page no.** |
|  | Allocation concealment mechanism | 18 | Mechanism used to implement the random allocation sequence (eg, central computer/telephone; sequentially numbered, opaque, sealed containers), describing any steps to conceal the sequence until interventions were assigned | 8-11 |
|  | Implementation | 19 | Whether the personnel who enrolled and those who assigned participants to the interventions had access to the random allocation sequence | 8-11 |
|  | Blinding | 20a | Who was blinded after assignment to interventions (eg, participants, care providers, outcome assessors, data analysts) | 8-9 |
|  |  | 20b | If blinded, how blinding was achieved and description of the similarity of interventions | 8-9 |
|  | Statistical methods | 21a | Statistical methods used to compare groups for primary and secondary outcomes, including harms | 13 |
|  |  | 21b | Definition of who is included in each analysis (eg, all randomised participants), and in which group | 11-13 |
|  |  | 21c | How missing data were handled in the analysis | 11-13 |
|  |  | 21d | Methods for any additional analyses (eg, subgroup and sensitivity analyses), distinguishing prespecified from post hoc | 13-14 |
|  | **Results** | | |  |
|  | Participant flow, including flow diagram | 22a | For each group, the numbers of participants who were randomly assigned, received intended intervention, and were analysed for the primary outcome | 14 |
|  |  | 22b | For each group, losses and exclusions after randomisation, together with reasons | 14 |
|  | Recruitment | 23a | Dates defining the periods of recruitment and follow-up for outcomes of benefits and harms | 15 |
|  |  | 23b | If relevant, why the trial ended or was stopped | - |
|  | Intervention and comparator delivery | 24a | Intervention and comparator as they were actually administered (eg, where appropriate, who delivered the intervention/comparator, how participants adhered, whether they were delivered as intended (fidelity)) | 15-16 |
|  |  | 24b | Concomitant care received during the trial for each group | 11-13 |
|  | Baseline data | 25 | A table showing baseline demographic and clinical characteristics for each group | 16 |
|  | Numbers analysed,  outcomes and estimation | 26 | For each primary and secondary outcome, by group:  ● the number of participants included in the analysis  ● the number of participants with available data at the outcome time point  ● result for each group, and the estimated effect size and its precision (such as 95% confidence interval)  ● for binary outcomes, presentation of both absolute and relative effect size | 16-21 |
|  | Harms | 27 | All harms or unintended events in each group | 16-21 |
|  | Ancillary analyses | 28 | Any other analyses performed, including subgroup and sensitivity analyses, distinguishing pre-specified from post hoc | 16-21 |
|  | **Discussion** | | | **22** |
|  | Interpretation | 29 | Interpretation consistent with results, balancing benefits and harms, and considering other relevant evidence | 22 |
|  | Limitations | 30 | Trial limitations, addressing sources of potential bias, imprecision, generalisability, and, if relevant, multiplicity of analyses | 24 |

Citation: Hopewell S, Chan AW, Collins GS, Hróbjartsson A, Moher D, Schulz KF, et al. CONSORT 2025 Statement: updated guideline for reporting randomised trials. BMJ. 2025; 388:e081123. <https://dx.doi.org/10.1136/bmj-2024-081123>
© 2025 Hopewell et al. This is an Open Access article distributed under the terms of the Creative Commons Attribution License (<https://creativecommons.org/licenses/by/4.0/>), which permits unrestricted use, distribution, and reproduction in any medium, provided the original work is properly cited.

*We strongly recommend reading this statement in conjunction with the CONSORT 2025 Explanation and Elaboration and/or the CONSORT 2025 Expanded Checklist for important clarifications on all the items. We also recommend reading relevant CONSORT extensions. See [www.consort-spirit.org](http://www.consort-spirit.org).

**REFERENCES**

1. Colado JC, Garcia-Masso X, Travis Triplett N, et al (2014) Construct and Concurrent Validation of a New Resistance Intensity Scale for Exercise with Thera-Band® Elastic Bands. J Sports Sci Med 13:758

2. Assumpção A, Matsutani LA, Yuan SL, et al (2018) Muscle stretching exercises and resistance training in fibromyalgia: which is better? A three-arm randomized controlled trial. Eur J Phys Rehabil Med 54:663–670. https://doi.org/10.23736/s1973-9087.17.04876-6
